# Plasma protein profiling of Mild Cognitive Impairment and Alzheimer’s disease using iTRAQ quantitative proteomics

**DOI:** 10.1186/1477-5956-12-5

**Published:** 2014-01-17

**Authors:** Fei Song, Anne Poljak, Nicole A Kochan, Mark Raftery, Henry Brodaty, George A Smythe, Perminder S Sachdev

**Affiliations:** 1 Centre for Healthy Brain Ageing (CHeBA), University of New South Wales, Sydney, Australia; 2 Bioanalytical Mass Spectrometry Facility, University of New South Wales, Sydney, Australia; 3 School of Medical Sciences, University of New South Wales, Sydney, Australia; 4 School of Psychiatry, University of New South Wales, Sydney, Australia

**Keywords:** Biomarkers, Isobaric tags for relative and absolute quantitation (iTRAQ), Plasma, Mild Cognitive Impairment, Alzheimer’s disease, Proteomics

## Abstract

**Background:**

With the promise of disease modifying treatments, there is a need for more specific diagnosis and prognosis of Alzheimer’s disease (AD) and mild cognitive impairment (MCI). Plasma biomarkers are likely to be utilised to increase diagnostic accuracy and specificity of AD and cognitive decline.

**Methods:**

Isobaric tags (iTRAQ) and proteomic methods were used to identify potential plasma biomarkers of MCI and AD. Relative protein expression level changes were quantified in plasma of 411 cognitively normal subjects, 19 AD patients and 261 MCI patients. Plasma was pooled into 4 groups including normal control, AD, amnestic single and multiple domain MCI (aMCI), and nonamnestic single and multiple domain MCI (nMCI). Western-blotting was used to validate iTRAQ data. Integrated function and protein interactions were explored using WEB based bioinformatics tools (DAVID v6.7 and STRING v9.0).

**Results:**

In at least two iTRAQ replicate experiments, 30 proteins were significantly dysregulated in MCI and AD plasma, relative to controls. These proteins included ApoA1, ApoB100, complement C3, C4b-binding protein, afamin, vitamin D-binding protein precursor, isoform 1 of Gelsolin actin regulator, Ig mμ chain C region (IGHM), histidine-rich glycoprotein and fibrinogen β and γ chains. Western-blotting confirmed that afamin was decreased and IGHM was increased in MCI and AD groups. Bioinformatics results indicated that these dysregulated proteins represented a diversity of biological processes, including acute inflammatory response, cholesterol transport and blood coagulation.

**Conclusion:**

These findings demonstrate that expression level changes in multiple proteins are observed in MCI and AD plasma. Some of these, such as afamin and IGHM, may be candidate biomarkers for AD and the predementia condition of MCI.

## Introduction

Alzheimer’s disease (AD) is the most common cause of dementia and approximately one in eight people over 65 years old are at risk. AD is an age-related and insidious-onset neurodegenerative disease [1]. Mild cognitive impairment (MCI) is proposed to be a phase of cognitive decline intermediate between normal health and dementia [2]. MCI patients may progress to AD, vascular disease and other kinds of dementia. The diagnosis of MCI and AD depends on a combination of clinical and neuropsychological tests, with no easy and effective diagnostic methods for use in the early stages of cognitive impairment. Early diagnosis may support measures to prevent disease progression from MCI to AD, and benefit the development of effective treatments. Several previous studies have focused on this field and reported some proteins as potential AD plasma biomarkers [3-5] and some literature review papers have also been published [6,7]. A few studies have chosen panels of specific proteins for analysis in the context of AD biomarkers [8,9], however few studies have applied discovery-based proteomics methods to the plasma of MCI subjects to identify biomarkers of the pre-dementia stage of AD.

Obtaining plasma is less invasive than other techniques such as lumbar puncture, and plasma is a body fluid widely accepted for use in clinical testing. It reflects the full complexity of body proteins in health and disease, containing biomarkers relevant for prediction, diagnosis or further investigation into the cause and effects of neurological disorders. Despite the ease in obtaining plasma, a major challenge associated with its analysis is its high complexity. The relatively high abundance of proteins like serum albumin and immunoglobulins, which together constitute more than 85% of the total protein content, masks the lower abundant proteins which may be potential biomarkers. To overcome this problem, the high abundance proteins must first be removed with the use of affinity depletion columns [10,11].

Our objective was to examine the alteration in global expression of proteins in the plasma of MCI and AD subjects in comparison with cognitively normal subjects. The iTRAQ approach was utilised to analyse immunodepleted plasma samples from amnestic single and multiple domain MCI (aMCI), nonamnestic single and multiple domain MCI (nMCI), AD and cognitively normal subjects to reveal candidate biomarkers. Pooled plasma from the various groups was assayed and the iTRAQ experiments repeated three times (three biological replicates), and run twice (two technical replicates) by two dimensional liquid chromatography (2D LC) tandem mass spectrometry (MS/MS). It was expected that studying plasma proteins in MCI and AD subjects using a discovery-based proteomics method might identify potential new protein biomarkers for neurodegenerative disease, and provide a screening tool for identifying specific proteins to study in more detail, as we have already begun to do [12].

## Experimental procedures

### Ethics statement and consent

Ethics committee approval was obtained from the University of New South Wales (UNSW) and the South East Sydney Area Health Service (SESAHS) ethics committees, and is in compliance with the Helsinki Declaration. Written informed consent was obtained from patients and, in the case of AD subjects, additionally from a significant family member.

### Subjects and samples

Plasma samples of aMCI (n = 147), nMCI (n = 114) and cognitively normal subjects (n = 411) were collected from the Wave 1 (baseline) of the Sydney Memory and Ageing Study (MAS) (n = 1073), a population based longitudinal study of non-demented older adults [13]. Plasma samples from AD patients (n = 19), who were volunteers for a drug trial of a cholinesterase inhibitor, were collected at the Memory Clinic of the Department of Old Age Psychiatry of the Prince of Wales Hospital. Patients met the NINCDS-ADRDA (National Institute of Neurological and Communicative Diseases and Stroke/Alzheimer’s Disease and Related Disorders Association) criteria [14] for clinically determined probable mild or moderate AD. Samples from all study groups were collected using the same protocol. Fasting plasma samples containing EDTA were aliquoted (100 μl) into polypropylene tubes, stored at −80°C, and only thawed immediately before assay.

### Clinical evaluation

The diagnosis of MCI used for this study was based on international consensus criteria [15] as follows: (a) complaint of decline in memory or other cognitive function which may be self- or informant-reported; (b) cognitive impairment on objective testing, i.e. not normal for age as determined by performance on at least one test measure 1.5 SDs or more below published normative values (or comparable standardised score compared to age and/or education-matched samples); (c) participants did not have a pre-existing diagnosis of dementia on entry to the study, had an adjusted mini–mental state examination (MMSE) score of ≥24 and did not meet DSM-IV (American Psychiatric Association Diagnostic and Statistical Manual, 4^th^ Edition) criteria [16] for possible or probable dementia; (d) essentially normal function or minimal impairment in IADLs defined by a total average score *<*3.0 on the informant rated B-ADL [17].

Two MCI subtypes [18] are defined according to the following cognitive impairment profiles: amnestic single and multiple domain (only memory domain impaired and memory plus at least one non-memory domain impaired), non-amnestic single and multiple domain (one non-memory domain impaired and more than one non-memory domain impaired). The criterion for impaired domain was met if at least one of the measures from the domain was impaired, with a score 1.5 SD below normative data. The full list of domains, tests used for measuring each domain, and the sources of normative data used to determine impairment is presented in a previously published paper [13].

### Sample preparation and immunodepletion

Individual plasma samples were pooled into four groups according to the diagnosis (Table 1), comprising cognitively normal, aMCI, nMCI and AD. Three aliquots (150 μl) of pooled plasma from each group were immunodepleted in each of three 5-plex iTRAQ experiments.

**Table 1:** Subject characteristics and iTRAQ reporter ion labelling

**Groups**	**Normal**	**aMCI**	**nMCI**	**AD**
Subject No.	411	147	114	19
Mean age (SD)	77.9 (4.5)	79.3 (4.9)	78.2 (4.3)	73.8 (8.0)
% male	42.8	57.1	37.7	68.4
iTRAQ tag	113	114	115	116

aMCI, amnestic single and multiple domain MCI.

nMCI, nonamnestic single and multiple domain MCI.

Six of the high abundance proteins in plasma were depleted using Multiple Affinity Removal Column (Hu-6, 4.6 × 50 mm, Agilent, Palo Alto, CA). These included albumin, IgG, antitrypsin, IgA, transferrin and haptoglobin. Manufacturer’s instructions were followed for all steps. This kit also contains buffers for sample loading, washing, eluting and regenerating the column. Prior to chromatography, plasma samples were diluted five-fold using Buffer A. Particulates from plasma were removed using 0.22 μm spin filter (Millipore, Bedford, MA). Diluted plasma (100 μl) was loaded for each chromatography step at a flow rate of 0.25 mL/min and immunodepletion was performed using an HP1090 HPLC system (Agilent, Sydney, Australia). The flow-through fraction containing low abundance proteins was collected between 1.50-6.50 minutes, and concentrated using Amicon Ultra centrifugal filter devices (Millipore, Billerica, MA). The filter device was spun at 5000 × g for 45 minutes at 4°C.

### Protein assay, protein reduction and alkylation

The total protein concentration of low abundance proteins from the immunodepletion column flow-through of each subject group was determined using the Bicinchoninic acid (BCA) total protein assay (Pierce, Rockford, IL). To remove potentially iTRAQ incompatible buffer components, a portion of each sample comprising 100 μg of total protein was precipitated for two hours in chilled acetone (neat) at −20°C. The protein pellet was then resuspended (20 μl dissolving buffer, 45.5 mM NaHCO_3_) and 1 μl denaturant SDS (2%) was added. Samples were then reduced with 2 μl 5 mM tris-(2-carboxyethyl) phosphine (TCEP) for one hour at 60°C, and alkylated with 200 mM iodoacetamide at ambient temperature for ten minutes to irreversibly block cysteine groups.

### Protein digestion and iTRAQ labelling

After reduction and alkylation, the proteins in each group were digested overnight with trypsin (4.44 μg/10 μL) at 37°C. Three biological replicates of each group were digested independently. Samples were then labelled with iTRAQ reagents following the protocol provided by the vendor (Applied Biosystems, Foster City, CA). Briefly, one vial of iTRAQ labelling reagent (dissolved in neat isopropanol) was used per subject group. The entire contents of each iTRAQ vial was added to each sample and incubated for two hours at 37°C. The pH was then measured and adjusted to 7.5 by adding dissolution buffer (45.5 mM NaHCO_3_). The iTRAQ labelled samples were then pooled into a single vial. The experimental design for iTRAQ reporter ion sample labelling is shown in Table 1.

All solid phase extraction (SPE) sample clean-up steps were carried out using a syringe pump (KD Scientific, Holliston, MA) at a flow rate of 9.5 mL/hr. iTRAQ labelled peptides were fractionated by strong cation exchange SPE (Applied Biosystems, Foster City, CA), dried under vacuum and passed through a C18 SPE cartridge to desalt the sample (Peptide MacroTrap, Michrom Bioresources, Auburn, CA), eluted with 500 μl CH_3_CN : water : formic acid (50:50:0.1, v:v:v), followed by 200 μl CH_3_CN. The flow-through from the C18 step was then passed through an Oasis SPE cartridge (Waters, Milford, MA) to maximise sample recovery by capturing any peptides which may have passed through the C18 SPE.

### Two-dimensional liquid chromatography and MS/MS analysis

2D LC was carried out according to a published approach [19,20]. Chromatography was carried out using an LC Packings capillary HPLC system (Dionex, Amsterdam, the Netherlands), comprising an *UltiMate* pump system, *Switchos* valve unit and *Famos* autosampler. A portion of the iTRAQ labelled peptide mixture (*ca* 5 μg) was injected onto a strong cation exchange micro column (0.75 × ~ 20 mm, Poros S10, Applied Biosystems, Foster City, CA) and eluted with 12 ammonium acetate elution steps (5, 10, 15, 20, 25, 30, 40, 50, 100, 250, 500 and 1000 mM). The eluent was captured onto a C18 pre-column cartridge (Michrom Bioresources, Auburn, CA). After a 10 min wash, the pre-column was switched in-line to a capillary column (10 cm) containing C18 reverse phase packing material (Magic, 5 μ, 200 Å, Michrom Bioresources, Auburn, CA). Peptides were eluted using a 75 min gradient of buffer A (H_2_O:CH_3_CN of 98:2 containing 0.1% formic acid-buffer) to buffer B (H_2_O:CH_3_CN of 20:80 containing 0.1% formic acid-buffer) at ~300 nL/min. High voltage (2300 V) was applied through a low volume tee (Upchurch Scientific, Oak Harbor, WA) at the column inlet and the outlet positioned approximately 1 cm from the orifice of an API QStar Elite hybrid tandem mass spectrometer (ABSciex, Forster City, CA). Positive ions were generated by electrospray ionisation (ESI) and the QStar operated in information-dependent acquisition (IDA) mode. A time-of-flight (TOF) MS survey scan was acquired (m/z 350–1700, 0.75 s) and the three largest multiply charged ions (counts > 20, charge state ≥ 2 and ≤ 4) sequentially selected by Q1 for MS/MS analysis. Nitrogen was used as collision gas and an optimum collision energy automatically chosen (based on charge state and mass). Tandem mass spectra were accumulated for up to 2.5 s (m/z 65–2000).

### Database searching, statistical analysis and bioinformatics

Protein identification and quantitation were performed using the MS/MS data (WIFF files) and the Paragon algorithm as implemented in Protein Pilot v2.0.1 software (Applied Biosystems/MDS Sciex, Foster City, CA). The database used was ipi. HUMAN. v 3.58. fasta [20]. Identification of proteins was only accepted with a ProteinPilot Unused Score of ≥1.3 (greater than 95% confidence interval). The Paragon method uses the miscleavage factor to calculate the probability of missed cleavages. Most commonly 1 or 2 missed cleavages are allowed. The ProteinPilot Biological Modifications option was selected to search for variable post-translational modifications (PTMs). This includes a list of >220 PTMs to include in the search. The modifications use the HUPO-PSI modification nomenclature. The only fixed modification used was iodoacetamide alkylation of cysteine residues. Mass tolerances were *ca* 50 ppm for the precursor and 0.2 Da for the fragment ion masses. Autobias correction was used to correct for systematic bias in sample pooling. Only proteins identified in all three iTRAQ experiments were further analysed. Quantitative data were exported into Excel (Microsoft, Bellevue, WA) for further analysis.

Integrated function and protein interactions were explored using Web-based bioinformatics tools: Database for Annotation, Visualisation and Integrated Discovery (DAVID v6.7) [21,22] and Search Tool for the Retrieval of Interacting Genes/Proteins (STRING v9) [23]. DAVID bioinformatics resources consist of an integrated biological knowledgebase and analytic tools aimed at systematically extracting biological meaning from large gene/protein lists. STRING is a meta-resource that aggregates most of the available information on protein-protein associations, scores and weights, and augments it with predicted interactions, as well as results of automatic literature-mining searches. The full set of dysregulated proteins in MCI and AD from iTRAQ results was entered into DAVID for functional analysis and STRING for the analysis of protein interaction. The background set for DAVID analysis was the full homosapiens genome (default set in DAVID).

### Western blot analysis

High abundance protein-depleted plasma samples were electrophoretically separated on a 1D NuPAGE 4-12% gradient gel (Invitrogen, Carlsbad, CA), with equal amounts of total protein (15 μg) loaded in each lane. Prestained markers were loaded in one lane to indicate approximate molecular weight. The gel was electroblotted (10 V, 80 mA, 2 hr) onto a nitrocellulose membrane in semidry transfer buffer (50 mM Tris HCl, 40 mM glycine, 1.3 mM SDS, 20% methanol, pH 9.2). Once the transfer was complete, the nitrocellulose membrane was incubated in skim milk powder solution (10% in 10 mM tris/HCl pH 7.5 containing 0.9% NaCl) containing the primary antibody. Immunoblotting was carried out with antibodies against afamin (0.86 μg/ml, Abcam, Cambridge, UK) and Ig mμ chain C region (Immunoglobulin heavy constant mu, IGHM) (1:200 dilution, Abnova Coporation, Taibei, Taiwan), followed by a secondary antibody (1:100,000 anti-rabbit IgG and anti-mouse IgG, respectively) (Pierce, Rockford, IL). Chemiluminescence blots were developed with 1:1 solution of Super Signal West Femto Luminol/Enhancer and Stable Peroxide buffer (Pierce, Rockford, IL) and using the manufacturer’s instructions. Chemiluminescence films were scanned using a KODAK Gel Logic 100 Imaging System (Sydney, Australia). Western blot images were then quantified using Labscan v3.00 software (Amersham Bioscience, UK).

## Results

### iTRAQ analysis of plasma proteins from aMCI, nMCI, AD and cognitively normal subjects to detect differential protein expression

Plasma protein expression profiles in MCI and AD were analysed by comparing the results of three biological replicates of the 5-plex iTRAQ experiment. Proteins identified in the three iTRAQ replicates and their reporter ion ratios relative to the control sample (reporter ion 113) are presented in Additional file 1: Table S1, Additional file 2: Table S2 and Additional file 3: Table S3. Protein Pilot v2.0 identified and quantified a total of 93, 107 and 96 proteins in the first, second and third biological replicates, respectively. The results of three iTRAQ replicates are summarised in Table 2.

**Table 2:** Summarized results of three iTRAQ experiments

**Biological replicate experiment**	Unused* (Conf) cutoff	**Number of unique proteins detected**	**Number of proteins before grouping**	**Number of distinct peptides identified**	**Number of spectra identified**	**% Total spectra ¶**
iTRAQ 1	>1.3 (95)	93	352	5047	11908	72.1
iTRAQ 2	>1.3 (95)	107	357	7614	17211	76.4
iTRAQ 3	>1.3 (95)	96	301	6392	15789	71.8

*A ProteinPilot Unused score of >1.3 indicates >95% confidence in correct protein sequence identification.

¶Spectra identified ÷ total MS/MS spectra.

In total, 145 unique proteins were identified, with a minimum unused score of ≥1.3 (which indicates a >95% confidence in correct sequence identification). A subset of 30 significantly dysregulated proteins (Table 3) were selected for further analysis using bioinformatics tools, the selection criteria being: 1) significant iTRAQ ratio alteration for a particular protein (p value ≤ 0.05) in at least one MCI subtype or AD group, and 2) consistent trends of significant alteration in at least two iTRAQ replicates. Thirty proteins met the criteria, and Table 3 shows the iTRAQ results of these proteins. Most ratios of dysregulated proteins were between 0.75 and 1.25. A substantial proportion (26.7%) of these proteins belonged to the apolipoprotein family. Protein level False Discovery Rate (FDR) analysis was carried out using ProteinPilot v4. The number of proteins detected at 5% FDR range from 93 to 111. Corresponding ProteinPilot confidence at 5% FDR ranges from 96.9% to 99.1%. Corresponding unused ProtScore at 5% FDR ranges from 1.51 to 2.04.

**Table 3:** Dysregulated proteins in the plasma of MCI subtypes and AD, identified in three iTRAQ biological replicate experiments

**Accession No.**	**Name (Acronym)**	**Replicate**	**aMCI: Normal**	**PVal**	**nMCI: Normal**^**a,b**^	**PVal**	**AD: Normal**	**PVal**
IPI00855916.1	Transthyretin (TTR)	1	**2.15**	0 ^***^	**1.15**	**0.05**	**1.57**	0 ^***^
		2	0.94	0.28	0.93	0.1	1.06	0.28
		3	0.91	0.13	**1.13**	0 ^***^	**1.19**	0 ^***^
IPI00745089.2	alpha 1B-glycoprotein precursor (A1BG)	2	**1.09**	**0.03**	**1.08**	**0.01**	**1.09**	**0.01**
		3	1	0.99	**1.06**	**0.02**	**1.17**	0 ^***^
IPI00477090.6	Ig mu chain C region (IGHM)	1	1.05	0.6	0.97	0.63	**1.41**	0 ^***^
		2	**1.48**	0 ^***^	**1.21**	**0.03**	**1.57**	0 ^***^
		3	**1.41**	0 ^***^	1	0.99	**1.47**	0 ^***^
IPI00022229.1	Apolipoprotein B-100 (APOB)	1	1.03	0.23	**1.31**^**b**^	0 ^***^	1.01	0.73
		2	0.99	0.63	**1.19**^**b**^	0 ^***^	1.01	0.56
		3	1.03	0.17	**1.03**^**b**^	**0.05**	1.02	0.18
IPI00032291.2	Complement C5 (C5)	1	1.04	0.5	**1.24**^**b**^	0 ^***^	0.99	0.82
		2	1	1	**1.14**^**b**^	0 ^***^	0.99	0.8
IPI00292530.1	Inter-alpha-trypsin inhibitor heavy chain H1 (ITIH1)	1	1.09	0.17	**1.3**^**b**^	**0.01**	1.05	0.25
		3	**1.24**	**0.01**	**1.13**^**b**^	**0.05**	1.05	0.27
IPI00026314.1	Isoform 1 of Gelsolin (GSN)	1	0.99	0.86	**1.13**^**b**^	**0.03**	1.04	0.41
		2	1.07	0.11	**1.14**^**b**^	0 ^***^	1.04	0.24
		3	**1.15**	0 ^***^	**1.09**^**b**^	**0.02**	0.95	0.31
IPI00019591.2	Complement factor B (CFB)	1	0.96	0.14	0.95	0.12	**0.95**	**0.05**
		2	**0.93**	**0.05**	**0.91**	0 ^***^	0.94	0.1
		3	0.96	0.29	**0.95**	**0.03**	**0.9**	0 ^***^
IPI00019943.1	Afamin (AFM)	1	0.94	0.32	**0.69**^**b**^	0 ^***^	0.95	0.25
		2	0.97	0.5	**0.83**^**b**^	0 ^***^	0.99	0.8
		3	**0.69**	0 ^***^	**0.87**^**b**^	0 ^***^	1.01	0.79
IPI00021727.1	C4b-binding protein alpha chain (C4BPA)	1	**0.93**	**0.02**	1.04	0.22	0.96	0.18
		2	**0.82**	**0.01**	0.98	0.77	0.97	0.56
		3	**0.85**	0 ^***^	**0.8**	**0.01**	**0.82**	0 ^***^
IPI00479116.1	Carboxypeptidase N subunit 2 (CPN2)	1	**0.79**	0 ^***^	**0.86**	**0.05**	**0.83**	**0.01**
		2	**0.57**	0 ^***^	1.01	0.88	**0.74**	0 ^***^
IPI00022371.1	Histidine-rich glycoprotein (HRG)	1	0.99	0.85	**0.66**^**b**^	0 ^***^	1.01	0.75
		2	1.1	0.07	**0.86**^**b**^	**0.02**	1.07	0.07
		3	**0.87**	**0.01**	0.95	0.15	0.96	0.2
IPI00006662.1	Apolipoprotein D (APOD)	1	0.99	0.92	1.03	0.67	**0.83**	**0.01**
		3	**0.71**	0 ^***^	1.05	0.18	1.06	0.19
IPI00478003.1	Alpha-2-macroglobulin (A2M)	1	0.99	0.42	0.98	0.21	**0.92**	0 ^***^
		2	**1.06**	0 ^***^	0.98	0.11	**1.04**	**0.02**
		3	**1.13**	0 ^***^	0.99	0.44	**0.9**	0 ^***^
IPI00021841.1	Apolipoprotein A-I (APOA1)	1	**1.38**	0 ^***^	**1.05**	**0.04**	**1.13**	0 ^***^
		2	**0.51**	0 ^***^	**0.81**	**0**	**0.63**	0 ^***^
		3	**0.66**	0 ^***^	1.03	0.07	**1.45**	0 ^***^
IPI00021854.1	Apolipoprotein A-II (APOA2)	1	**1.31**	0 ^***^	**0.81**	**0.01**	**0.85**	0 ^***^
		2	**0.5**	0 ^***^	**0.91**	**0.05**	**0.81**	0 ^***^
		3	**0.53**	0 ^***^	1.06	0.13	0.98	0.68
IPI00304273.2	Apolipoprotein A-IV (APOA4)	1	**1.21**	0 ^***^	0.99	0.8	0.97	0.39
		2	**0.72**	0 ^***^	0.96	0.28	**0.83**	0 ^***^
		3	**0.67**	0 ^***^	0.99	0.85	**1.11**	0 ^***^
IPI00021856.3	Apolipoprotein C-II (APOC2)	1	**1.49**	**0.05**	1.07	0.68	1.01	0.96
		2	**0.55**	**0.01**	**0.76**	**0.02**	**0.61**	**0.01**
		3	**0.42**	**0.02**	0.7	0.16	0.97	0.92
IPI00298828.3	Beta-2-glycoprotein 1 (APOH)	1	1.01	0.91	0.95	0.23	**0.89**	**0.05**
		2	**1.15**	0 ^***^	0.97	0.43	**1.18**	0 ^***^
		3	**0.8**	0 ^***^	0.97	0.5	**0.87**	**0.01**
IPI00783987.2	Complement C3 (C3)	1	0.97	0.07	**0.94**	0 ^***^	**0.93**	0 ^***^
		2	1.01	0.64	**0.95**	0 ^***^	0.98	0.24
		3	**1.14**	0 ^***^	1.01	0.24	**0.92**	0 ^***^
IPI00400826.1	Clusterin isoform 1 (CLU)	1	1.1	0.16	1	0.99	1.05	0.36
		2	0.92	0.13	0.93	0.07	1.01	0.91
		3	**0.81**	**0.02**	**0.92**	**0.04**	**1.14**	**0.02**
IPI00021885.1	Isoform 1 of Fibrinogen alpha chain (FGA)	1	**0.94**	0 ^***^	**0.93**	0 ^***^	1	0.88
		2	1.01	0.76	**0.89**	0 ^***^	**1.07**	0 ^***^
		3	**1.15**	0 ^***^	**0.95**	**0.01**	1.01	0.76
IPI00298497.3	Fibrinogen beta chain (FGB)	1	0.97	0.15	0.98	0.31	1.01	0.71
		2	1.01	0.79	**0.92**	0 ^***^	**1.05**	**0.01**
		3	**1.16**	0 ^***^	**0.96**	**0.05**	**0.96**	**0.04**
IPI00877792.1	Fibrinogen gamma chain (FGG)	1	**1.1**	0 ^***^	0.99	0.65	**1.09**	0 ^***^
		2	**0.85**	0 ^***^	**0.88**	0 ^***^	**0.91**	0 ^***^
		3	1.01	0.78	**0.91**	0 ^***^	**0.92**	0 ^***^
IPI00742696.2	vitamin D-binding protein precursor (GC)	1	1.01	0.83	1	0.88	1.04	0.19
		2	**0.27**	0 ^***^	**0.44**	0 ^***^	**0.27**	0 ^***^
		3	**0.68**	0 ^***^	0.98	0.44	**1.07**	**0.04**
IPI00641737.1	Haptoglobin-related protein (HPR)	1	**0.87**	0 ^***^	**1.08**	**0.03**	0.95	0.23
		2	**1.1**	0 ^***^	**1.06**	**0.03**	**1.06**	**0.05**
		3	1.07	0.11	0.96	0.27	1.01	0.77
IPI00896413.1	Inter-alpha-trypsin inhibito heavy chain H4 (ITIH4)	1	**1.12**	**0.04**	0.77	0.08	0.9	0.21
		2	0.94	0.14	0.97	0.25	**0.94**	**0.05**
		3	0.96	0.24	0.95	0.08	**0.93**	**0.02**
IPI00884926.1	Orosomucoid 1 precursor (ORM1)	2	**0.85**	0 ^***^	1.03	0.37	0.92	0.1
		3	**0.59**	0 ^***^	1	0.98	**1.07**	**0.05**
IPI00550991.3	Alpha-1-antichymotrypsin (SERPINA3)	1	**1.21**	0 ^***^	1.05	0.15	**1.17**	0 ^***^
		2	**0.83**	0 ^***^	**0.93**	**0.05**	**0.87**	0 ^***^
		3	0.99	0.91	1	0.98	**1.11**	0 ^***^
IPI00879231.1	Alpha-2-antiplasmin (SERPINF2)	2	0.89	0.25	**1.25**	0 ^***^	1	0.99
		3	**0.86**	**0.05**	**1.21**	**0.01**	1.05	0.53

^**a**^This column represents nonamnestic MCI, with either single or multiple domains affected. The data are single and multiple domain unless otherwise indicated. ^**b**^Nonamnestic multiple domain MCI (nmdMCI) data. Boldface data, P<0.05. ***P < 0.001. aMCI, amnestic single and multiple domain MCI. nMCI, nonamnestic single and multiple domain MCI. The IPI human database was used during ProteinPilot processing.

### Annotation and functional enrichment of dysregulated proteins; DAVID v6.7

For an overview of the dysregulated proteins in MCI and AD, functional analysis including molecular functions, biological process and pathways were performed using DAVID v6.7 tools (http://david.abcc.ncifcrf.gov/).

Molecular function analysis revealed that 29% of the proteins dysregulated in MCI and AD had enzyme inhibitor activity, 17% had cell surface binding, and other functions included enzyme activator activity and sterol transport activity. Biological process analysis showed the dysregulated proteins are most often involved in the acute inflammatory response, lipid transport, blood coagulation, cell activation and complement activation (Table 4).

**Table 4:** Functional classification of dysregulated proteins in mild cognitive impairment and Alzheimer’s disease using DAVID bioinformatics tool

		**Enrichment score¶**	**Protein count**	**P value**	**Benjamin**
Biological process	Acute inflammatory response	9.6	8	2.5E-11	6.1E-9
	Blood coagulation	4.19	4	3.1E-4	5.2E-3
	Cell activation	4.19	4	1.6E-2	3.3E-2
	Lipid transport	3.35	5	3.4E-5	1.7E-3
	Complement activation	2.45	3	6.6E-4	8.9E-3
Molecular function	Enzyme inhibitor activity	6.66	8	1.5E-8	3.5E-7
	Cell surface binding	4.19	5	3.6E-8	5.0E-7
	Enzyme activator activity	3.35	4	7.4E-3	3.7E-2
	Sterol transporter activity	3.35	3	1.3E-4	9.0E-4
	Unclassified		7		

¶The full human genome was used as the reference list [21,22]. The classification stringency was set to medium.

KEGG pathway analysis indicated that one significant pathway of complement and blood coagulation was present. Seven proteins are involved in this pathway including A2M, complement component 3, complement component 4 binding protein, fibrinogen α chain (FGA), fibrinogen beta chain (FGB), fibrinogen γ chain (FGG) and alpha-2-antiplasmin.

### Protein networks; STRING v9.0

**Figure 1:**
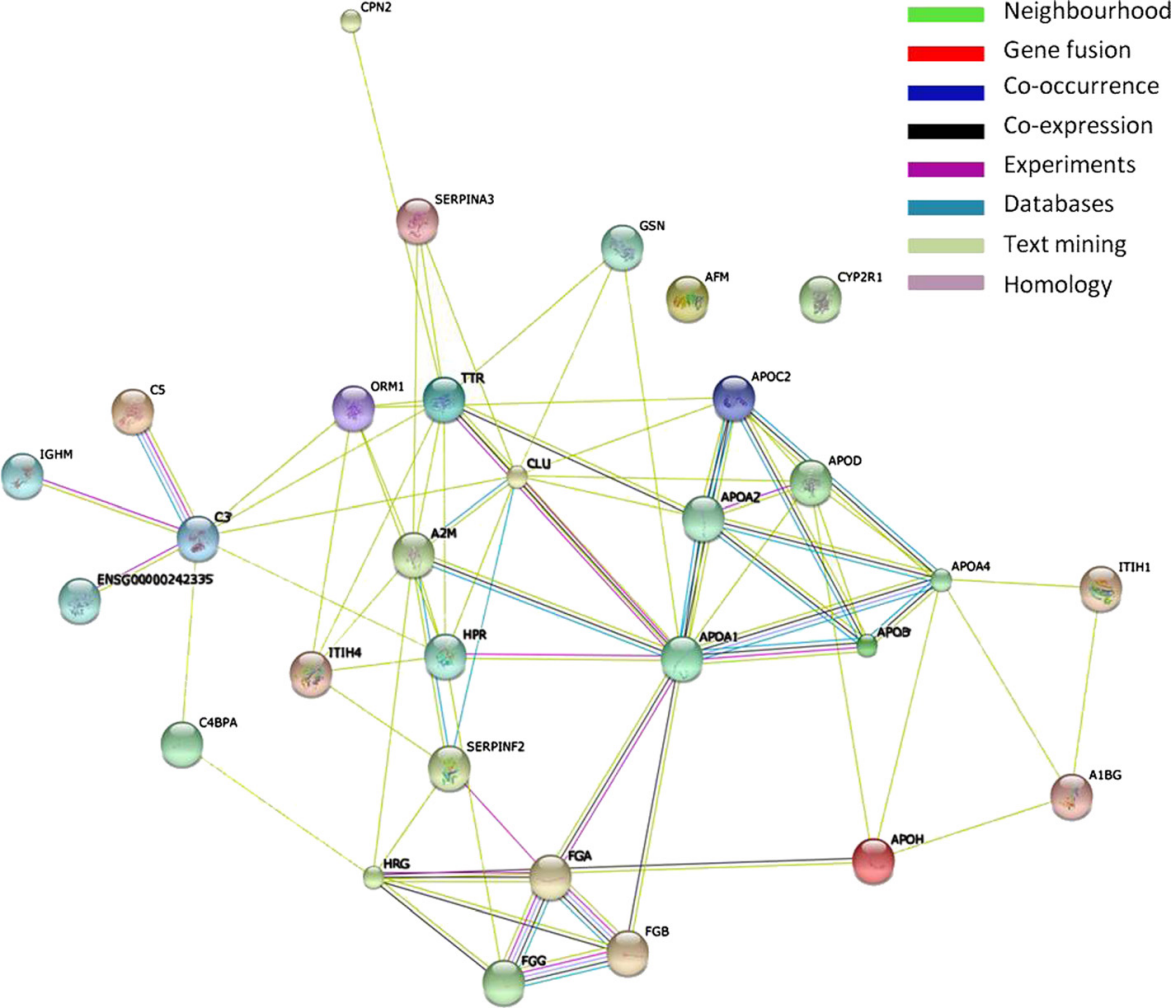
Association networks of dysregulated proteins using STRING v9.0.

Figure 1 shows the protein-protein interaction networks generated by database and web-tool STRING 9.0 (http://string-db.org). The 28 dysregulated proteins (see acronyms in Table 3) determined from the iTRAQ experiment were analysed using this molecular interaction tool. Two functional modules are apparent in the network, forming tightly connected clusters (Figure 1). The first functional module includes apolipoprotein A1(ApoA1), apolipoprotein A2(ApoA2), apolipoprotein B (ApoB), apolipoprotein A4 (ApoA4), apolipoprotein D (ApoD) and apolipoprotein C2 (ApoC2); the second includes histidine-rich glycoprotein (HRG), FGA, FGB, FGG. The highest numbers of connectivities are to ApoA1 and ApoA2.

### Western blot results

**Figure 2:**
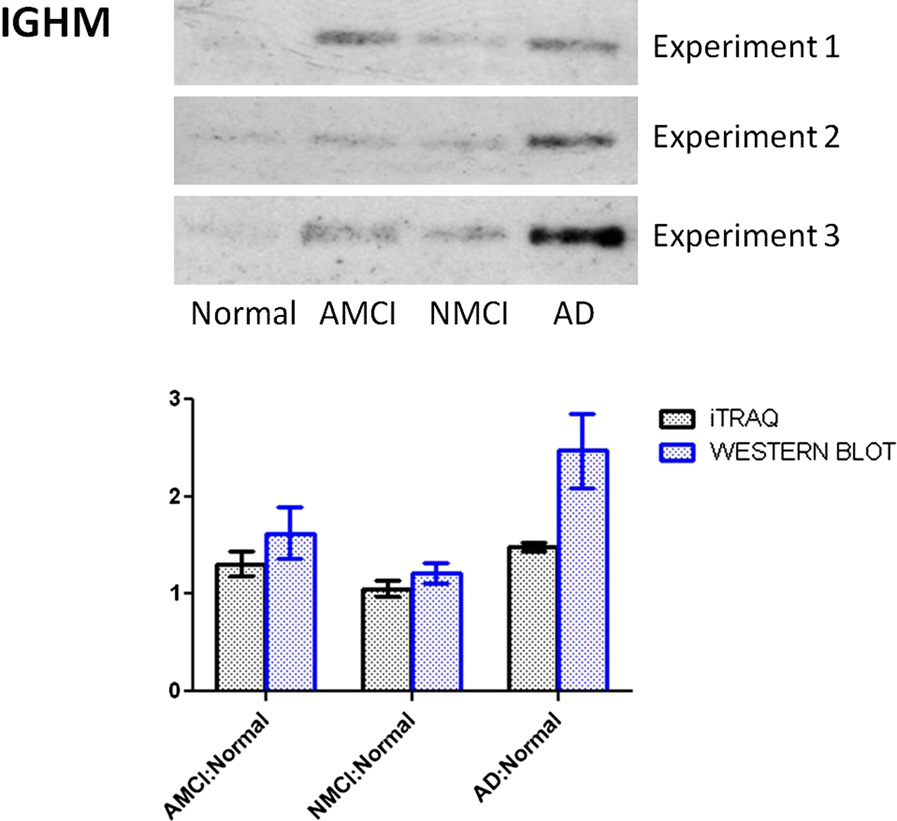
Comparison of western blot analysis and iTRAQ results for IGHM.

**Figure 3:**
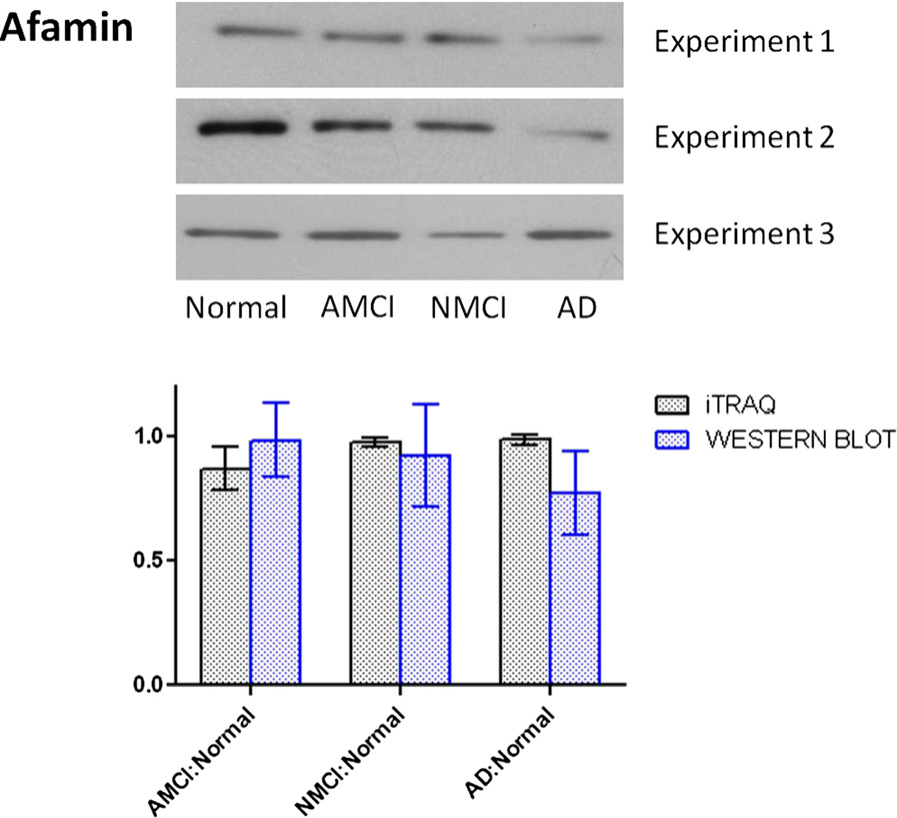
Comparison of western blot analysis and iTRAQ results for afamin.

Three replicates of western blot experiments were performed to provide an independent comparison and validation of the iTRAQ experiments. One upregulated (IGHM) and one downregulated (afamin) protein were chosen for this comparison, and the reasons were (i) they are two of the most significantly deregulated proteins in our list, with ratios of up to 1.57 for IGHM and down to 0.69 for afamin), (ii) they have not previously been studied in MCI and AD, and (iii) they may provide new insight into the mechanism of disease. Western blot results confirmed that IGHM was increased in MCI and AD groups (Figure 2), and afamin was decreased in MCI and AD group (Figure 3). Figures 2 and 3 also show the comparison of western blot and iTRAQ outcomes of IGHM and afamin, and confirm consistency of up regulation (ratio >1) or down regulation (ratio <1) between the two experimental approaches, despite slight differences between the mean values.

### Validation in individual samples

A group of apolipoproteins, including ApoA1, ApoA2, ApoB, ApoH, ApoJ, have been selected from iTRAQ results and measured in individual samples, and the data published [12]. Table 5 compares the average ratios of these 5 apolipoproteins between disease and normal groups in the iTRAQ pooled plasma samples and individual validation based on previous multiplex assay data, using the same MAS Wave 1 study group [21]. These two results were consistent and showed that the ApoA1, ApoA2, ApoH were downregulated and ApoB and ApoJ were upregulated in the MCI group.

**Table 5:** Comparison of average disease/normal iTRAQ ratios for apolipoproteins on pooled plasma with individual samples assayed by ELISA multiplex assays

iTRAQ*				**Multiplex assay§**
	**aMCI:Normal**	**nMCI:Normal**	**AD:Normal**	**MCI:Normal**
ApoA1	0.85	0.96	1.07	0.91
ApoA2	0.78	0.93	0.88	0.94
ApoB	1.02	1.18	1.01	1.06
ApoH	0.99	0.96	0.98	0.91
ApoJ	0.94	1.10	1.07	1.10

*average ratios of three iTRAQ replicates.

§ratios of the mean values of apolipoprotein levels assayed in MCI and Normal groups using individual plasma samples, as detailed in Song *et al.*[12].

## Discussion

Quantitation of serum or plasma proteins using iTRAQ analysis has recently been suggested as a suitable approach for the detection of biomarkers [24]. In a preliminary proof-of-principle experiment, this current study assessed whether this method could be suitable for the detection of biomarkers useful for MCI and AD screening. We identified 30 proteins for which expression levels, relative to normal controls, were significantly altered in pooled MCI and AD plasma samples. Bioinformatics results indicated that these dysregulated proteins represented a diversity of biological functions, including immunity and inflammation, transportation of important regulatory biomolecules, blood coagulation and cell processes. Protein association network analysis also revealed dysregulation of a sizeable group of apolipoproteins and fibrinogen chains with important biological functions.

### Dysregulated proteins in MCI and AD

A large number of dysregulated proteins in our iTRAQ results are apolipoproteins, and bioinformatic results showed that they are highly associated with other dysregulated proteins. Recently, animal model and clinical studies have also suggested that apolipoproteins are involved in neurodegenerative processes in AD [25-28]. Several apolipoproteins were therefore selected for further validation in individual plasma samples using multiplex analysis. These results have been published [12].

In this study, FGA, FGB and FGG were identified as dysregulated proteins in MCI and AD. STRING protein connections also revealed them to be components of a functional module. Fibrin is the primary protein component of a blood clot. Its inactive precursor, fibrinogen, circulates in the blood as a large complex molecule of 340 kDa. Under normal circumstances, fibrinogen is excluded from the brain by the blood–brain barrier, but it has been found to accumulate in the extravascular space over time as AD pathology progresses [29-31]. Elevated fibrinogen levels are reported to be associated with increased risk for AD and dementia [32,33]. An *in vitro* study recently suggested that the interaction between Aβ and fibrinogen modifies fibrinogen’s structure which may then lead to abnormal fibrin clot formation and vascular abnormalities in AD [34].

Consistently up-regulated proteins observed in the MCI and/or AD group(s) and in all three biological replicate runs include IGHM, inter-alpha-trypsin inhibitor heavy chain H1(ITIH1), transthyretin (TTR) and gelsolin. ITIH1 is one of the heavy chains of a serine protease inhibitor that may serve to carry hyaluronan in plasma, and plays a role in inflammation and carcinogenesis [35]. Some research suggested that ITIH1 may be related to the incidence of Bipolar disorder [36] and human solid tumors [35]. The role of ITIH1 in MCI and AD has not been fully studied. TTR is a carrier protein which transports thyroid hormones in the plasma and CSF, and also transports retinol in the plasma. In the CNS, TTR also interacts with Aβ, and it has been suggested that it protects against Aβ deposition [37]. One recent study reported the opposite results, finding that TTR levels in plasma were significantly lower in AD subjects [38]. Gelsolin is a cytoskeletal protein that presents both intracellularly and extracellularly, and also binds to Aβ and inhibits the fibrillization of Aβ [39,40]. One study showed that plasma gelsolin levels were decreased in AD subjects [41]. Only a handful of studies examined TTR and gelsolin levels in AD, and so far no definite conclusion can be drawn. More research, especially large population-based studies, is needed in this area.

There were 6 consistently down-regulated proteins of MCI and/or AD group in three biological runs, including C4b-binding protein alpha chain (C4BPA), complement factor B (CFB), apolipoprotein D (ApoD), afamin, carboxypeptidase N subunit 2 (CPN2) and histidine-rich glycoprotein (HRG). Along with a single beta chain, seven C4BPA assemble into the predominant isoform of C4b-binding protein (C4BP), a complement inhibitor that controls activation of the classical pathway of complement activation. C4BP accumulates in Aβ plaques of AD brains [42-44], and binds with Aβ1-42 peptide [43]. CFB is a component of the alternative pathway of complement activation. Our results support the possibility that complement regulatory proteins are involved in the pathogenesis of AD. ApoD is localised in Aβ plaques of AD brains [45]. Some studies have shown that ApoD expression is dysregulated in AD brains [46-48]. Carboxypeptidase N (CPN) is a zinc metalloprotease in plasma, containing two enzymatically active small subunits (CPN1) and two large subunits (CPN2) [49]. As a major regulator of inflammation, CPN cleaves carboxy-terminal arginines and lysines from peptides such as complement anaphylatoxins (C3a, C5a), kinins and creatine kinase [50]. However, the role of CPN in AD is not clear. HRG is involved in fibrinolysis and coagulation [51], and also plays a role in inflammation and immunity [52]. The role of HRG in AD has not been fully investigated, and only one study reported that HRG was decreased in AD sera [53].

The age of our AD group was slightly younger than the MCI group and there were more male patients in the AD group. This may be a limitation of this study, but the impact of disease on the proteomic results is probably more significant than the age and sex. In our previously published work [12], which aimed to validate iTRAQ apolipoprotein results in individual samples, differences in apolipoprotein expression level withstood covariance for age and sex, as well as several other population variables such as years of education, APOE ϵ4 carrier status, and hypolipidaemic medications [12]. Moreover, the ratios of some proteins identified in this study are not consistent in all three biological replicates. This may be due to the modest ratio changes of some of these proteins in MCI and AD. However using other quantitative methods to measure this group of proteins in large samples may be promising, and the iTRAQ method has proved to be a useful screening tool.

### Afamin and cognitive decline

It was found in this study that the oxidative stress-associated protein afamin was down-regulated in pooled MCI and AD plasma using both iTRAQ and western blot analysis. To our knowledge, this is the first report of afamin downregulation in MCI and AD plasma. Human plasma afamin (α-albumin, α1T-glycoprotein) is the fourth member of the albumin gene family that also includes albumin, α -fetoprotein and vitamin D-binding protein. Interestingly, like afamin, vitamin-D binding protein is also downregulated in our list of iTRAQ deregulated proteins (Table 3). A recent study revealed that afamin is synthesised in the endothelial cells of the blood–brain barrier (BBB) and plays a role in vitamin E transport in an *in vitro* model of the BBB [54]. Vitamin E belongs to the family of nonenzymatic antioxidants and is bound to afamin, its specific carrier protein in plasma and extravascular fluids [55,56]. Vitamin E is an important fat-soluble antioxidant and plays a crucial role in protecting against oxidative damage and disease [57]. Oxidative stress has been implicated in the inflammatory reaction seen in AD brain. New gene chip technology demonstrates that vitamin E deficiency can have a strong impact on gene expression in the hippocampus [58], one of the earliest brain regions to be affected in AD.

To date the role of dietary vitamin intake in AD has been inconclusive, although it is generally accepted that levels of plasma antioxidants, including vitamin levels, are lower in AD [59-61]. Some clinical studies suggest that Vitamin E intake might delay AD progression [62]. The Chicago Health and Ageing Project showed that increased dietary vitamin E intake (not from vitamin supplements) correlated with lowered AD risk [63]. In the Rotterdam Study, individuals who reported higher intakes of vitamins C and E at baseline had a lower incidence of AD [64]. Consequently this area deserves more research attention, particularly with the use of large scale population based studies.

Furthermore, several other proteomic studies have identified afamin as a potential biomarker in other disorders, including simian immunodeficiency virus (SIV) induced central nervous system disease [10], ovarian cancer [65], congenital disorders of glycosylation [66] and Down syndrome [67]. Thus alterations in afamin are not specific to neurodegenerative disease and quantitative changes are observed in other disorders as well. However, from the standpoint of clinical use, afamin could be integrated with other measures in a potentially multipronged approach to diagnostic, prognostic and aetiologic studies. The specificity of afamin might be improved in a test which includes multiple markers, and this kind of “multiplexed” approach may be necessary in complex multifactorial conditions/diseases such as MCI and AD.

### IGHM and cognitive decline

IGHM gained our attention because it had relatively high fold changes in MCI and AD groups in comparison with other dysregulated proteins (Table 3). Both iTRAQ and western blot data showed that IGHM increased in MCI and AD relative to normal plasma. Our results are consistent with published data about the activation of the immune system in AD. Upregulation of inflammation in AD and MCI is recognised both in the CNS and in plasma [68]. A typical immunoglobulin is composed of two identical heavy chains and two identical light chains joined by disulfide bonds. Each Ig heavy chain has an N-terminal variable region containing the antigen-binding site and a C-terminal constant (C) region. The IGHM gene encodes the C region of the mu heavy chain, which defines the IgM isotype. Human natural anti-Aβ antibodies are present in both IgG and IgM repertoire [69]. Aβ binding antibodies are present in healthy humans and AD patients [70,71]. IgM anti-Aβ antibodies showed higher specific activity for Aβ than IgG anti- Aβ antibody [69], and the ability to clear cerebral Aβ without entering the brain of a mouse model of AD [72]. One recent study found that the levels of plasma Aβ1-42 autoantibodies in patients with MCI that progressed to AD, were significantly higher than in cognitively normal controls, but not so for MCI stable cases [73]. Aβ antibody titers were negatively correlated with cognitive status such that more cognitively impaired individuals tended to exhibit higher anti-Aβ IgG titers [71]. Development of immunotherapeutic reagents for AD is based on the expression of specific proteolytic activity by naturally occurring immunoglobulins [74], and hydrolysis of peripheral Aβ consequent to their capture by IgMs may induce increased Aβ clearance from the brain [75].

In conclusion, iTRAQ was chosen as a screening tool in this study, to identify interesting candidate proteins as biomarkers of MCI and AD. The function and interaction of dysregulated proteins was studied using bioinformatics tools. 28 proteins were found dysregulated in plasma of MCI and AD. Bioinformatic results showed the dysregulated proteins are most often involved in the acute inflammatory response, cholesterol transport, blood coagulation and complement activation. Western blot was used to further validate the change of afamin and IGHM in plasma of MCI and AD. iTRAQ and western blot results both confirmed that afamin was down-regulated and IGHM was up-regulated in MCI and AD. These preliminary results suggest that plasma is a rich source for studying plasma biomarkers of MCI and AD and that iTRAQ proteomics is an excellent screening and discovery tool. The data presented here includes proteins which have not previously been studied in the context of MCI or AD, and may represent excellent targets for a multiplexed biomarker assay.

## Abbreviations

AD: Alzheimer’s disease; MCI: Mild cognitive impairment; iTRAQ: Isobaric tags for relative and absolute quantitation; MCI aMCI: Amnestic single and multiple domain; MCI nMCI: Nonamnestic single and multiple domain; IGHM: Ig mu chain C region; MAS: Sydney memory and ageing study; NINCDS-ADRDA: National Institute of Neurological and Communicative Diseases and Stroke/Alzheimer’s Disease and Related Disorders Association; MMSE: Mini–mental state examination; DAVID v6.7: Database for annotation, visualisation and integrated discovery; STRING v9: Search tool for the retrieval of interacting genes/proteins; MS/MS: Two dimensional liquid chromatography; 2D LC: Tandem mass spectrometry; FGA: Fibrinogen alpha chain; FGB: Fibrinogen beta chain; FGG: Fibrinogen gamma chain; ApoA1: Apolipoprotein A1; ApoA2: Apolipoprotein A2; ApoB: Apolipoprotein B; ApoA4: Apolipoprotein A4; ApoD: Apolipoprotein D; ApoC2: Apolipoprotein C2; HRG: Histidine-rich glycoprotein; ITIH1: Inter-alpha-trypsin inhibitor heavy chain H1; TTR: Transthyretin; C4BPA: C4b-binding protein alpha chain; CFB: Complement factor B; ApoD: Apolipoprotein D; CPN2: Carboxypeptidase N subunit 2; HRG: Histidine-rich glycoprotein.

## Competing interests

The authors declare that they have no competing interests.

## Authors’ contribution

FS carried out iTRAQ experiments and data analysis, and drafted the manuscript. AP and PS conceived and supervised experimental work and data analysis, and edited the manuscript. NK was responsible for clinical diagnosis. MR provided technical support of experiments and instruments. HB and GS were involved in manuscript editing. All authors read and approved the final manuscript.

## Supplementary Material

Additional file 1: Table S1 The results of total identified proteins in iTRAQ experiment 1. Click here for file

Additional file 2: Table S2 The results of total identified proteins of iTRAQ experiment 2. Click here for file

Additional file 3: Table S3 The results of total identified proteins of iTRAQ experiment 3. Click here for file
