# Supplementary material for: Plasma protein profiling of Mild Cognitive Impairment and Alzheimer’s disease using iTRAQ quantitative proteomics
Source: Proteome Sci. 2014 Jan 17;12:5. doi: 10.1186/1477-5956-12-5 (PMC3898732; doi:10.1186/1477-5956-12-5)
Supplement: Additional file 1: Table S1 — The results of total identified proteins in iTRAQ experiment 1. [file 1477-5956-12-5-S1.docx]

Supplementary table 1 The results of total identified proteins in iTRAQ experiment 1

| N | Unused | Total | % Cov | Accession # | Name | Peptide (95%) | aMCI:  Normal | nMCI:  Normal | AD:  Normal | nmdMCI:  Normal |
| --- | --- | --- | --- | --- | --- | --- | --- | --- | --- | --- |
| 1 | 190.42 | 190.42 | 83.8 | IPI:IPI00783987.2 | Complement C3 | 134 | 0.97* | 0.94* | 0.93* | 1.16* |
| 2 | 190 | 190 | 71.3 | IPI:IPI00022229.1 | Apolipoprotein B-100 | 88 | 1.03 | 0.98 | 1.01 | 1.31* |
| 3 | 157.22 | 157.22 | 77.8 | IPI:IPI00478003.1 | Alpha-2-macroglobulin | 135 | 0.99 | 0.98 | 0.92* | 1.07* |
| 4 | 118.25 | 118.25 | 87 | IPI:IPI00745872.2 | Isoform 1 of Serum albumin | 173 | --- | --- | --- | --- |
| 5 | 97.26 | 97.29 | 65.9 | IPI:IPI00887154.2 | Complement component 4B | 55 | 1.42 | 1.05 | 0.96 | 2.44 |
| 6 | 70.39 | 70.39 | 83.9 | IPI:IPI00298497.3 | Fibrinogen beta chain | 74 | 0.97 | 0.98 | 1.01 | 1.13* |
| 7 | 66.51 | 66.51 | 64.7 | IPI:IPI00029739.5 | Isoform 1 of Complement factor H | 37 | 0.99 | 1.03 | 1.01 | 1.01 |
| 8 | 64.83 | 64.83 | 66.4 | IPI:IPI00021885.1 | Isoform 1 of Fibrinogen alpha chain | 76 | 0.94* | 0.93* | 1.00 | 1.06 |
| 9 | 54.07 | 54.08 | 80.4 | IPI:IPI00877792.1 | Fibrinogen gamma chain | 60 | 1.10* | 0.99 | 1.09* | 1.27* |
| 10 | 51.51 | 51.51 | 96.6 | IPI:IPI00021841.1 | Apolipoprotein A-I | 48 | 1.38* | 1.05* | 1.13* | 1.70* |
| 11 | 48.27 | 48.27 | 67.1 | IPI:IPI00022488.1 | Hemopexin | 42 | 1.04 | 1.03 | 1.07* | 0.95 |
| 12 | 48.13 | 48.13 | 60 | IPI:IPI00017601.1 | Ceruloplasmin | 29 | 1.00 | 0.99 | 0.97 | 0.86* |
| 13 | 47.25 | 47.26 | 54.9 | IPI:IPI00019591.2 | Complement factor B | 26 | 0.96 | 0.95 | 0.95* | 1.10* |
| 14 | 46.91 | 47.17 | 63.7 | IPI:IPI00019580.1 | Plasminogen | 20 | 0.94* | 1.00 | 1.00 | 0.86* |
| 15 | 43.47 | 43.47 | 73.9 | IPI:IPI00896419.2 | Inter-alpha-trypsin inhibitor heavy chain H4 | 22 | 0.96 | 1.14 | 1.09 | 0.84 |
| 16 | 43.13 | 43.13 | 76.4 | IPI:IPI00742696.2 | Vitamin D-binding protein precursor | 20 | 1.01 | 1.00 | 1.04 | 0.72* |
| 17 | 42.7 | 42.7 | 73.5 | IPI:IPI00304273.2 | Apolipoprotein A-IV | 22 | 1.21* | 0.99 | 0.97 | 1.08 |
| 18 | 40.82 | 40.82 | 52.9 | IPI:IPI00305461.2 | Inter-alpha-trypsin inhibitor heavy chain H2 | 22 | 0.96 | 1.01 | 1.05 | 1.07 |
| 19 | 35.26 | 35.26 | 60.5 | IPI:IPI00021727.1 | C4b-binding protein alpha chain | 18 | 0.93* | 1.04 | 0.96 | 1.14* |
| 20 | 34.13 | 34.13 | 64.5 | IPI:IPI00641737.1 | HPR 47 kDa protein | 17 | 0.87* | 1.08* | 0.95 | 0.90 |
| 21 | 33.57 | 33.57 | 68.9 | IPI:IPI00797833.3 | Kininogen 1 | 18 | 0.96 | 0.95 | 0.93 | 0.95 |
| 22 | 29.48 | 29.48 | 64.6 | IPI:IPI00019568.1 | Prothrombin | 15 | 1.00 | 0.93 | 0.97 | 1.01 |
| 23 | 28.47 | 28.48 | 54.4 | IPI:IPI00292530.1 | Inter-alpha-trypsin inhibitor heavy chain H1 | 15 | 1.09 | 1.08 | 1.05 | 1.30* |
| 24 | 28.17 | 28.18 | 58.3 | IPI:IPI00032179.2 | Antithrombin III variant | 13 | 1.18 | 0.89 | 1.03 | 1.26 |
| 25 | 27.78 | 27.78 | 60 | IPI:IPI00550991.3 | Alpha-1-antichymotrypsin | 17 | 1.21* | 1.05 | 1.17* | 1.60* |
| 26 | 24.06 | 24.06 | 63.9 | IPI:IPI00400826.1 | Clusterin isoform 1 | 12 | 1.10 | 1.00 | 1.05 | 1.32* |
| 27 | 23.72 | 23.73 | 37.6 | IPI:IPI00032291.2 | Complement C5 | 8 | 1.04 | 0.98 | 0.99 | 1.24* |
| 28 | 23.3 | 23.3 | 70.1 | IPI:IPI00745089.2 | 1B-glycoprotein precursor | 11 | 0.95 | 0.98 | 1.06 | 0.90 |
| 29 | 22.99 | 22.99 | 59.4 | IPI:IPI00298828.3 | Beta-2-glycoprotein 1 | 12 | 1.01 | 0.95 | 0.89* | 0.80* |
| 30 | 22.39 | 22.39 | 59.4 | IPI:IPI00022431.2 | Alpha-2-HS-glycoprotein | 15 | 1.01 | 1.00 | 1.06 | 0.83* |
| 31 | 21.24 | 21.24 | 50.6 | IPI:IPI00022395.1 | Complement component C9 | 10 | 1.09 | 1.18 | 1.10 | 1.00 |
| 32 | 18.71 | 18.71 | 57.5 | IPI:IPI00879931.1 | Plasma protease C1 inhibitor | 10 | 1.26* | 1.06 | 1.12* | 1.35* |
| 33 | 18.43 | 18.43 | 52.4 | IPI:IPI00026314.1 | Isoform 1 of Gelsolin | 9 | 0.99 | 1.00 | 1.04 | 1.13* |
| 34 | 18.13 | 18.25 | 62.1 | IPI:IPI00019943.1 | Afamin | 7 | 0.94 | 1.01 | 0.95 | 0.69* |
| 35 | 17.86 | 17.86 | 48.6 | IPI:IPI00022371.1 | Histidine-rich glycoprotein | 9 | 0.99 | 0.99 | 1.01 | 0.66* |
| 36 | 16.84 | 16.84 | 49.6 | IPI:IPI00298971.1 | Vitronectin | 9 | 0.98 | 0.98 | 1.01 | 0.91 |
| 37 | 16.53 | 16.53 | 82.1 | IPI:IPI00022429.3 | Alpha-1-acid glycoprotein 1 | 15 | 1.17* | 1.04 | 1.12* | 1.11 |
| 38 | 15.93 | 15.93 | 67.3 | IPI:IPI00480192.1 | Retinol binding protein 4, plasma | 10 | 1.02 | 1.07 | 0.87* | 0.69* |
| 39 | 15.39 | 15.39 | 51 | IPI:IPI00292950.4 | Serpin peptidase inhibitor, clade D (Heparin cofactor), member 1 | 6 | 0.93 | 1.00 | 1.00 | 1.03 |
| 40 | 13.97 | 13.97 | 74.4 | IPI:IPI00021842.1 | Apolipoprotein E | 6 | 1.25* | 0.94 | 0.99 | 1.39* |
| 41 | 13.94 | 13.94 | 69 | IPI:IPI00855916.1 | Transthyretin | 13 | 2.15* | 1.15* | 1.57* | 1.44* |
| 42 | 11.94 | 11.94 | 46.5 | IPI:IPI00749179.2 | Putative uncharacterized protein C1S | 5 | 0.96 | 0.97 | 1.01 | 0.96 |
| 43 | 10.98 | 10.98 | 37.4 | IPI:IPI00291867.3 | Complement factor I | 5 | 0.94 | 0.91 | 0.95 | 0.81 |
| 44 | 10.13 | 10.13 | 52.9 | IPI:IPI00006114.4 | Pigment epithelium-derived factor | 5 | 0.86 | 0.93 | 0.89 | 0.92 |
| 45 | 10.11 | 10.11 | 48.1 | IPI:IPI00879231.1 | Alpha-2-antiplasmin | 4 | 0.92 | 0.95 | 1.01 | 1.09 |
| 46 | 10 | 10 | 50.6 | IPI:IPI00022394.2 | Complement C1q subcomponent subunit C | 5 | 0.96 | 0.92 | 1.04 | 0.77 |
| 47 | 9.91 | 9.92 | 59.1 | IPI:IPI00166729.4 | Alpha-2-glycoprotein 1, zinc | 4 | 0.98 | 0.98 | 1.05 | 0.76* |
| 48 | 9.8 | 9.8 | 43.5 | IPI:IPI00022426.1 | Protein AMBP | 5 | 0.95 | 0.92 | 1.01 | 1.06 |
| 49 | 9.62 | 9.62 | 36.9 | IPI:IPI00296165.6 | Complement C1r subcomponent | 4 | 1.04 | 1.00 | 1.07 | 1.17 |
| 50 | 9.48 | 9.48 | 49.8 | IPI:IPI00924574.1 | Apolioportein D | 6 | 0.99 | 1.03 | 0.83* | 0.78* |
| 51 | 9.29 | 9.29 | 41 | IPI:IPI00032220.3 | Angiotensinogen | 4 | 1.03 | 1.09 | 1.06 | 1.01 |
| 52 | 8.59 | 8.59 | 37.4 | IPI:IPI00879709.3 | Complement component 6 precursor | 3 | 0.88 | 1.06 | 0.88 | 0.93 |
| 53 | 8.58 | 8.58 | 32.7 | IPI:IPI00218732.3 | Serum paraoxonase/arylesterase 1 | 4 | 1.02 | 1.05 | 1.09 | 0.99 |
| 54 | 8.49 | 8.49 | 47 | IPI:IPI00021854.1 | Apolipoprotein A-II | 10 | 1.31* | 0.81* | 0.85* | 0.88 |
| 55 | 8.44 | 8.44 | 60.9 | IPI:IPI00020986.2 | Lumican | 4 | 1.13 | 1.04 | 1.06 | 0.90 |
| 56 | 8.25 | 8.28 | 41.5 | IPI:IPI00654888.4 | Plasma kallikrein | 4 | 0.85* | 0.89 | 0.95 | 0.72* |
| 57 | 7.8 | 7.8 | 47.1 | IPI:IPI00873445.1 | PROS1 80 kDa protein | 4 | 1.08 | 1.12 | 1.05 | 1.13 |
| 58 | 7.44 | 7.44 | 37.8 | IPI:IPI00296608.6 | Complement component C7 | 4 | 0.94 | 1.01 | 0.91 | 0.85 |
| 59 | 7.31 | 7.31 | 38.3 | IPI:IPI00477090.6 | IGHM protein | 3 | 1.05 | 0.97 | 1.41* | 1.02 |
| 60 | 6.98 | 6.98 | 49.8 | IPI:IPI00022391.1 | Serum amyloid P-component | 3 | 0.92* | 0.98 | 1.02 | 0.85 |
| 61 | 6.71 | 21.56 | 57.9 | IPI:IPI00844156.2 | SERPINC1 protein | 9 | 1.03 | 1.04 | 1.04 | 0.92 |
| 62 | 6 | 6 | 50.5 | IPI:IPI00009028.1 | Tetranectin | 3 | 1.00 | 0.81 | 0.72 | 0.98 |
| 63 | 5.53 | 5.53 | 55.4 | IPI:IPI00019399.1 | Serum amyloid A-4 protein | 3 | 1.00 | 0.99 | 0.91 | 0.96 |
| 64 | 5.27 | 5.27 | 55.8 | IPI:IPI00643948.2 | Complement component 1, q subcomponent, B chain | 2 | 0.85 | 0.98 | 1.06 | 0.96 |
| 65 | 4.81 | 4.81 | 50.2 | IPI:IPI00293925.2 | Isoform 1 of Ficolin-3 | 2 | 0.84* | 0.98 | 0.91 | 0.93 |
| 66 | 4.59 | 4.79 | 24.8 | IPI:IPI00746623.2 | Hyaluronan-binding protein 2 | 2 | 1.09 | 1.19 | 1.08 | 1.01 |
| 67 | 4.3 | 4.3 | 33.9 | IPI:IPI00011252.1 | Complement component C8 alpha chain | 2 | 0.98 | 0.90 | 0.92 | 0.98 |
| 68 | 4.28 | 8.57 | 64.2 | IPI:IPI00020091.1 | Alpha-1-acid glycoprotein 2 | 6 | 1.10 | 1.12 | 1.06 | 0.78 |
| 69 | 4.2 | 9.96 | 50.9 | IPI:IPI00011264.2 | Complement factor H-related protein 1 | 5 | 0.55 | 1.26 | 0.85 | 0.41 |
| 70 | 4.08 | 4.08 | 33.7 | IPI:IPI00021856.3 | Apolipoprotein C-II | 2 | 1.49* | 1.07 | 1.01 | 1.24 |
| 71 | 4.01 | 4.01 | 52.1 | IPI:IPI00410714.5 | HBA1 Hemoglobin subunit alpha | 3 | 0.88 | 0.86 | 0.68* | 1.03 |
| 72 | 4 | 4 | 50.4 | IPI:IPI00657670.1 | Apolipoprotein C-III variant 1 | 4 | 1.00 | 0.90 | 0.74 | 0.91 |
| 73 | 3.86 | 3.87 | 34.2 | IPI:IPI00294395.1 | Complement component C8 beta chain | 1 | 1.06 | 1.09 | 0.91 | 1.02 |
| 74 | 3.79 | 3.79 | 74.4 | IPI:IPI00514397.1 | Apolipoprotein M | 2 | 0.97 | 0.96 | 0.69* | 0.88 |
| 75 | 3.71 | 3.71 | 35.4 | IPI:IPI00884107.1 | Beta-globin gene from a thalassemia patient | 2 | 0.85 | 0.92 | 0.66 | 0.92 |
| 76 | 3.53 | 3.53 | 46.1 | IPI:IPI00022392.1 | Complement C1q subcomponent subunit A | 2 | 0.83 | 0.87* | 0.91 | 0.74 |
| 77 | 3.05 | 3.05 | 30.3 | IPI:IPI00855785.1 | Isoform 15 of Fibronectin | 1 | 0.79 | 0.93 | 1.10 | 0.74* |
| 78 | 3.01 | 3.01 | 17.9 | IPI:IPI00296534.2 | Isoform D of Fibulin-1 | 1 | 1.12 | 1.20 | 1.08 | 1.38* |
| 79 | 3 | 3 | 39.3 | IPI:IPI00328609.3 | Kallistatin | 1 | 1.25 | 1.32 | 1.33* | 1.72 |
| 80 | 2.92 | 2.92 | 36 | IPI:IPI00513935.1 | Complement component 8, gamma polypeptide | 1 | 0.92 | 0.84 | 0.94* | 1.07 |
| 81 | 2.38 | 2.38 | 39.1 | IPI:IPI00163207.1 | Isoform 1 of N-acetylmuramoyl-L-alanine amidase | 1 | 0.92 | 0.98 | 0.84 | 0.73 |
| 82 | 2.31 | 2.51 | 39.8 | IPI:IPI00029168.1 | Apolipoprotein(a) | 1 | 0.76 | 0.87* | 0.50* | 1.10 |
| 83 | 2.01 | 2.01 | 41.8 | IPI:IPI00329775.7 | Isoform 1 of Carboxypeptidase B2 | 1 | 0.96 | 1.09 | 0.87* | 0.66* |
| 84 | 2 | 42.37 | 73.1 | IPI:IPI00896413.1 | ITIH4 100 kDa protein | 22 | 1.12* | 0.77 | 0.90 | 1.37* |
| 85 | 2 | 2 | 45.2 | IPI:IPI00807459.1 | IGKC protein | 1 | 0.98 | 1.04 | 1.48 | 0.78 |
| 86 | 2 | 2 | 27.5 | IPI:IPI00019576.1 | Coagulation factor X | 1 | 0.68 | 0.40 | 0.89 | 0.81 |
| 87 | 1.98 | 1.98 | 25.9 | IPI:IPI00479116.1 | Carboxypeptidase N subunit 2 | 0 | 0.79* | 0.86* | 0.83* | 0.89 |
| 88 | 1.65 | 1.65 | 39.8 | IPI:IPI00022445.1 | Platelet basic protein | 1 | 0.74 | 0.80 | 0.66 | 0.38 |
| 89 | 1.58 | 1.58 | 39.1 | IPI:IPI00607784.1 | Isoform 3 of Zinc finger protein 638 | 1 | 1.22 | 0.64 | 0.84 | 0.87 |
| 90 | 1.56 | 1.56 | 33.8 | IPI:IPI00019581.1 | Coagulation factor XII | 0 | 0.94 | 0.89* | 0.90* | 0.89 |
| 91 | 1.52 | 1.56 | 63.1 | IPI:IPI00006146.4 | SAA2 serum amyloid A2 isoform a | 1 | 0.96 | 0.90 | 0.61 | 1.12 |
| 92 | 1.37 | 1.37 | 25.3 | IPI:IPI00152868.5 | Probable phospholipid-transporting ATPase IM | 0 | 0.91 | 1.07 | 1.06 | 1.80 |
| 93 | 1.3 | 1.7 | 47 | IPI:IPI00003400.1 | Leukocyte antigen CD37 | 1 | --- | --- | --- | --- |

- P<0.05

Down-regulation (The ratios of proteins in disease groups are significantly less than 1.)

Up-regulation (The ratios of proteins in disease groups are significantly more than 1.)
