# Supplementary material for: Plasma protein profiling of Mild Cognitive Impairment and Alzheimer’s disease using iTRAQ quantitative proteomics
Source: Proteome Sci. 2014 Jan 17;12:5. doi: 10.1186/1477-5956-12-5 (PMC3898732; doi:10.1186/1477-5956-12-5)
Supplement: Additional file 2: Table S2 — The results of total identified proteins of iTRAQ experiment 2. [file 1477-5956-12-5-S2.docx]

Supplementary table 2 The results of total identified proteins of iTRAQ experiment 2

| N | Unused | Total | % Cov | Accession # | Name | Peptide (95%) | aMCI:  Normal | nMCI:  Normal | AD:  Normal | nmdMCI:  Normal |
| --- | --- | --- | --- | --- | --- | --- | --- | --- | --- | --- |
| 1 | 228.21 | 228.21 | 95.20 | IPI:IPI00783987.2 | Complement C3 | 219 | 1.01 | 0.95* | 0.98 | 1.12* |
| 2 | 221.92 | 221.92 | 80.80 | IPI:IPI00022229.1 | Apolipoprotein B-100 | 118 | 0.99 | 1.02 | 1.01 | 1.19* |
| 3 | 196.50 | 196.50 | 99.00 | IPI:IPI00745872.2 | Isoform 1 of Serum albumin | 342 | --- | --- | --- | --- |
| 4 | 193.11 | 193.11 | 89.60 | IPI:IPI00478003.1 | Alpha-2-macroglobulin | 224 | 1.06* | 0.98 | 1.04* | 1.11* |
| 5 | 118.01 | 118.01 | 89.50 | IPI:IPI00643525.1 | Putative uncharacterized protein C4A | 85 |  |  |  |  |
| 6 | 90.34 | 90.34 | 98.20 | IPI:IPI00298497.3 | Fibrinogen beta chain | 122 | 1.01 | 0.92* | 1.05* | 1.06* |
| 7 | 74.13 | 74.13 | 76.00 | IPI:IPI00029739.5 | Isoform 1 of Complement factor H | 49 | 1.01 | 0.98 | 1.00 | 1.03 |
| 8 | 72.13 | 72.13 | 80.40 | IPI:IPI00021885.1 | Fibrinogen alpha chain | 93 | 1.01 | 0.89* | 1.07* | 1.09* |
| 9 | 69.52 | 69.52 | 77.70 | IPI:IPI00017601.1 | Ceruloplasmin | 47 | 0.96 | 0.89* | 1.00 | 1.03 |
| 10 | 61.09 | 61.09 | 84.80 | IPI:IPI00022488.1 | Hemopexin | 69 | 0.98 | 1.00 | 1.05 | 1.08* |
| 11 | 60.86 | 60.86 | 71.20 | IPI:IPI00019591.2 | Complement factor B | 36 | 0.93 | 0.91* | 0.94 | 1.09* |
| 12 | 59.95 | 59.95 | 87.20 | IPI:IPI00877792.1 | Fibrinogen gamma chain | 88 | 0.85* | 0.88* | 0.91* | 1.12* |
| 13 | 56.40 | 56.40 | 85.40 | IPI:IPI00742696.2 | Vitamin D-binding protein precursor | 36 | 0.27* | 0.44* | 0.27* | 0.39* |
| 14 | 53.09 | 53.09 | 97.40 | IPI:IPI00021841.1 | Apolipoprotein A-I | 51 | 0.51* | 0.81* | 0.63* | 0.91* |
| 15 | 50.43 | 50.43 | 82.70 | IPI:IPI00896419.2 | Inter-alpha-trypsin inhibitor heavy chain H4 | 31 | 0.94 | 0.97 | 0.94 | 1.01 |
| 16 | 46.86 | 46.86 | 91.20 | IPI:IPI00641737.1 | Haptoglobin-related protein | 26 | 1.10* | 1.06* | 1.06 | 1.02 |
| 17 | 46.74 | 46.90 | 73.30 | IPI:IPI00019580.1 | Plasminogen | 24 | 1.01 | 0.95 | 0.96 | 0.94 |
| 18 | 46.64 | 46.64 | 91.10 | IPI:IPI00215894.1 | Isoform LMW of Kininogen-1 | 26 | 0.99 | 0.97 | 1.06 | 1.22 |
| 19 | 42.15 | 42.15 | 78.80 | IPI:IPI00298828.3 | Beta-2-glycoprotein 1 | 32 | 1.15* | 0.97 | 1.18* | 0.89* |
| 20 | 40.79 | 40.79 | 80.30 | IPI:IPI00305461.2 | Inter-alpha-trypsin inhibitor heavy chain H2 | 24 | 1.07* | 1.00 | 1.08* | 1.01 |
| 21 | 39.57 | 39.57 | 91.90 | IPI:IPI00847179.1 | Apolipoprotein A-IV precursor | 21 | 0.72* | 0.96 | 0.83* | 0.95 |
| 22 | 35.72 | 35.72 | 85.20 | IPI:IPI00032179.2 | Antithrombin III variant | 24 | 0.85* | 0.87 | 0.87* | 1.01 |
| 23 | 33.04 | 33.05 | 64.30 | IPI:IPI00032291.2 | Complement C5 | 14 | 1.00 | 1.04 | 0.99 | 1.14* |
| 24 | 31.80 | 31.80 | 78.00 | IPI:IPI00019568.1 | Prothrombin | 19 | 0.89 | 0.99 | 0.91 | 1.11* |
| 25 | 31.41 | 31.41 | 78.60 | IPI:IPI00745089.2 | Alpha 1B-glycoprotein precursor | 22 | 1.09* | 1.08* | 1.09* | 1.02 |
| 26 | 31.06 | 31.06 | 89.50 | IPI:IPI00550991.3 | Alpha-1-antichymotrypsin | 20 | 0.83* | 0.93* | 0.87* | 1.06 |
| 27 | 30.57 | 30.57 | 85.30 | IPI:IPI00926249.1 | AHSG 39 kDa protein | 25 | 0.97 | 0.93* | 0.96 | 0.98 |
| 28 | 28.55 | 28.56 | 75.90 | IPI:IPI00292530.1 | Inter-alpha-trypsin inhibitor heavy chain H1 | 19 | 0.99 | 0.95 | 0.99 | 1.04 |
| 29 | 26.62 | 26.62 | 54.90 | IPI:IPI00022371.1 | Histidine-rich glycoprotein | 16 | 1.10 | 0.93 | 1.07 | 0.86* |
| 30 | 24.40 | 24.40 | 77.40 | IPI:IPI00400826.1 | Clusterin isoform 1 | 11 | 0.92 | 0.93 | 1.01 | 1.13* |
| 31 | 23.91 | 23.91 | 85.30 | IPI:IPI00855916.1 | Transthyretin | 28 | 0.94 | 0.93 | 1.06 | 1.19* |
| 32 | 22.93 | 22.93 | 77.20 | IPI:IPI00026314.1 | Isoform 1 of Gelsolin | 11 | 1.07 | 0.94 | 1.04 | 1.14* |
| 33 | 22.91 | 22.91 | 66.60 | IPI:IPI00291866.5 | Plasma protease C1 inhibitor | 14 | 0.80* | 0.88* | 0.85 | 1.02 |
| 34 | 20.62 | 20.62 | 67.40 | IPI:IPI00292950.4 | D1 Serpin peptidase inhibitor, clade D (Heparin cofactor), member 1 | 10 | 0.95 | 1.00 | 0.97 | 1.15* |
| 35 | 20.53 | 20.53 | 99.50 | IPI:IPI00884926.1 | Orosomucoid 1 precursor | 33 | 0.85* | 1.03 | 0.92 | 0.91 |
| 36 | 20.32 | 20.32 | 63.10 | IPI:IPI00022395.1 | Complement component C9 | 11 | 1.02 | 1.09 | 0.99 | 1.13* |
| 37 | 17.58 | 17.58 | 66.20 | IPI:IPI00022426.1 | Protein AMBP | 9 | 1.11 | 1.00 | 1.08 | 1.08 |
| 38 | 17.05 | 17.05 | 84.40 | IPI:IPI00844536.2 | Retinol binding protein 4, plasma, isoform CRA_b | 13 | 1.06 | 0.95 | 1.09 | 0.95 |
| 39 | 16.98 | 16.98 | 59.50 | IPI:IPI00296165.6 | Complement C1r subcomponent | 7 | 1.01 | 0.96 | 0.92 | 1.02 |
| 40 | 16.81 | 16.98 | 59.30 | IPI:IPI00019943.1 | Afamin | 5 | 0.97 | 0.95 | 0.99 | 0.83* |
| 41 | 16.50 | 16.50 | 54.00 | IPI:IPI00298971.1 | Vitronectin | 16 | 1.02 | 0.91* | 1.00 | 0.97 |
| 42 | 14.17 | 14.17 | 67.80 | IPI:IPI00166729.4 | Alpha-2-glycoprotein 1, zinc | 7 | 0.96 | 1.03 | 0.98 | 0.82 |
| 43 | 14.00 | 14.00 | 60.00 | IPI:IPI00291867.3 | Complement factor I | 7 | 1.00 | 0.93* | 1.01 | 1.02 |
| 44 | 13.85 | 13.85 | 57.30 | IPI:IPI00749179.2 | Putative uncharacterized protein C1S | 7 | 1.00 | 0.88 | 1.01 | 0.98 |
| 45 | 13.71 | 13.71 | 57.40 | IPI:IPI00022391.1 | Serum amyloid P-component | 6 | 1.08 | 0.98 | 1.12* | 1.07 |
| 46 | 13.19 | 13.19 | 50.10 | IPI:IPI00021727.1 | C4b-binding protein alpha chain | 5 | 0.82* | 0.98 | 0.97 | 1.30* |
| 47 | 12.72 | 12.72 | 81.40 | IPI:IPI00021842.1 | Apolipoprotein E | 6 | 0.92 | 1.05 | 0.88 | 1.05 |
| 48 | 12.14 | 12.16 | 56.60 | IPI:IPI00654888.4 | Plasma kallikrein | 6 | 1.17 | 1.16 | 1.12 | ­ |
| 49 | 11.18 | 11.19 | 48.40 | IPI:IPI00855785.1 | Isoform 15 of Fibronectin | 5 | 0.81* | 0.90 | 1.08 | 0.99 |
| 50 | 11.03 | 11.03 | 74.00 | IPI:IPI00020986.2 | Lumican | 5 | 0.74* | 0.96 | 0.81 | 0.90 |
| 51 | 10.87 | 10.87 | 71.50 | IPI:IPI00218732.3 | Serum paraoxonase/arylesterase 1 | 5 | 1.06 | 1.10* | 1.16* | 1.11* |
| 52 | 10.49 | 10.49 | 62.20 | IPI:IPI00329775.7 | Isoform 1 of Carboxypeptidase B2 | 5 | 1.04 | 1.00 | 0.89 | 1.03 |
| 53 | 10.20 | 10.20 | 69.50 | IPI:IPI00032220.3 | Angiotensinogen | 5 | 0.83 | 0.89 | 0.84 | 0.99 |
| 54 | 10.18 | 10.18 | 63.20 | IPI:IPI00394992.1 | Isoform 2 of N-acetylmuramoyl-L-  alanine amidase | 5 | 1.17 | 1.29* | 1.59 | 1.16 |
| 55 | 9.81 | 9.81 | 65.30 | IPI:IPI00006114.4 | Pigment epithelium-derived factor | 4 | 0.94 | 1.01 | 1.04 | 1.06 |
| 56 | 9.72 | 9.72 | 70.00 | IPI:IPI00021854.1 | Apolipoprotein A-II | 13 | 0.50* | 0.91* | 0.81* | 0.84* |
| 57 | 9.33 | 9.33 | 49.50 | IPI:IPI00879709.3 | Complement component 6 precursor | 2 | 1.22* | 1.02 | 1.10 | 1.22* |
| 58 | 9.06 | 9.06 | 69.10 | IPI:IPI00328609.3 | Kallistatin | 5 | 0.95 | 0.95 | 1.13 | 1.03 |
| 59 | 8.21 | 8.21 | 44.60 | IPI:IPI00296608.6 | Complement component C7 | 4 | 1.05 | 0.97 | 0.96 | 1.15 |
| 60 | 8.08 | 8.08 | 68.60 | IPI:IPI00022394.2 | Complement C1q subcomponent subunit C | 4 | 1.07 | 0.98 | 1.06 | 1.07 |
| 61 | 7.55 | 20.78 | 81.90 | IPI:IPI00844156.2 | SERPINC1 protein | 11 | 0.81 | 0.72* | 0.81 | 0.62* |
| 62 | 7.22 | 7.22 | 55.50 | IPI:IPI00294395.1 | Complement component C8 beta chain | 3 | 1.02 | 1.03 | 1.02 | 1.15 |
| 63 | 7.21 | 7.21 | 79.20 | IPI:IPI00643948.2 | Complement component 1, q subcomponent, B chain | 7 | 1.11 | 1.04 | 1.06 | 1.10 |
| 64 | 7.04 | 11.48 | 97.50 | IPI:IPI00020091.1 | Alpha-1-acid glycoprotein 2 | 9 | 0.83* | 0.93 | 0.89 | 0.78* |
| 65 | 7.00 | 7.00 | 66.80 | IPI:IPI00011261.2 | Complement component C8 gamma chain | 3 | 1.14 | 1.05 | 1.09 | 1.11 |
| 66 | 6.95 | 26.22 | 64.70 | IPI:IPI00025426.2 | Isoform 1 of Pregnancy zone protein | 21 | 0.77* | 0.78 | 0.70 | 0.94 |
| 67 | 6.87 | 6.87 | 53.80 | IPI:IPI00549291.4 | IGHM protein | 4 | 1.48* | 1.21* | 1.57* | 1.36* |
| 68 | 6.65 | 6.65 | 51.30 | IPI:IPI00879231.1 | Alpha-2-antiplasmin | 3 | 0.89 | 1.25* | 1.00 | 1.32* |
| 69 | 6.08 | 6.09 | 72.20 | IPI:IPI00293925.2 | Isoform 1 of Ficolin-3 | 3 | 1.28* | 1.13 | 1.24* | 1.05 |
| 70 | 5.96 | 5.96 | 70.00 | IPI:IPI00019399.1 | Serum amyloid A-4 protein | 3 | 0.96 | 1.13 | 1.18* | 1.12* |
| 71 | 5.32 | 5.32 | 76.70 | IPI:IPI00006662.1 | Apolipoprotein D | 2 | 0.97 | 1.12 | 1.15 | 0.98 |
| 72 | 5.25 | 5.25 | 65.80 | IPI:IPI00007221.1 | Plasma serine protease inhibitor | 2 | 0.91 | 0.94 | 0.77 | 0.85 |
| 73 | 4.73 | 4.83 | 34.30 | IPI:IPI00746623.2 | Hyaluronan-binding protein 2 | 2 | 1.02 | 0.83* | 1.06 | 0.96 |
| 74 | 4.52 | 5.74 | 64.10 | IPI:IPI00292946.1 | Thyroxine-binding globulin | 2 | 0.78 | 1.07 | 0.88 | 1.00 |
| 75 | 4.45 | 4.45 | 42.80 | IPI:IPI00019581.1 | Coagulation factor XII | 1 | 1.16 | 1.19 | 1.16 | 0.99 |
| 76 | 4.35 | 117.06 | 89.20 | IPI:IPI00654875.1 | Complement C4-B | 84 | 0.97 | 1.82 | 1.02 | 1.06 |
| 77 | 4.08 | 4.08 | 34.10 | IPI:IPI00027235.1 | Isoform 1 of Attractin | 1 | 1.22* | 1.15 | 1.15 | 1.21 |
| 78 | 4.02 | 4.02 | 52.30 | IPI:IPI00514475.5 | Apolipoprotein L1 | 2 | 0.79 | 1.38 | 0.87* | 0.51 |
| 79 | 4.00 | 4.00 | 53.90 | IPI:IPI00011252.1 | Complement component C8 alpha chain | 2 | 1.02 | 1.05 | 1.32* | 1.33 |
| 80 | 4.00 | 4.00 | 39.40 | IPI:IPI00853068.1 | HBA1 Alpha 2 globin variant (Fragment) | 3 | 1.01 | 1.08 | 0.92 | 1.13 |
| 81 | 4.00 | 4.00 | 62.40 | IPI:IPI00657670.1 | Apolipoprotein C-III variant 1 | 6 | 1.12 | 1.02 | 1.27* | 0.93 |
| 82 | 3.17 | 3.17 | 55.30 | IPI:IPI00784985.1 | IGK@ protein | 1 | 1.58* | 0.89 | 1.83* | 1.36 |
| 83 | 3.12 | 3.12 | 30.70 | IPI:IPI00021856.3 | Apolipoprotein C-II | 1 | 0.55* | 0.76* | 0.61* | 0.52 |
| 84 | 2.96 | 3.05 | 53.10 | IPI:IPI00218949.1 | Isoform Short of Complement factor H-related protein 2 | 1 | 0.93 | 1.01 | 1.13 | 0.90 |
| 85 | 2.64 | 2.64 | 59.90 | IPI:IPI00654755.3 | Hemoglobin subunit beta | 2 | 0.90 | 1.03 | 0.77* | 1.06 |
| 86 | 2.54 | 2.54 | 49.60 | IPI:IPI00873445.1 | PROS1 80 kDa protein | 1 | 0.76 | 1.47* | 1.21 | 1.33* |
| 87 | 2.24 | 2.24 | 74.20 | IPI:IPI00925635.1 | Insulin-like growth factor binding protein, acid labile subunit isoform 1 precursor | 1 | 0.80 | 1.30 | 0.99 | 1.08 |
| 88 | 2.11 | 7.60 | 56.10 | IPI:IPI00303963.1 | Complement C2 | 4 | 1.00 | 0.87 | 0.88 | 0.96 |
| 89 | 2.04 | 44.68 | 84.50 | IPI:IPI00032328.2 | Isoform HMW of Kininogen-1 | 25 | 0.95 | 0.69 | 0.87 | 0.83 |
| 90 | 2.02 | 2.02 | 43.30 | IPI:IPI00744685.3 | Biotinidase | 1 | 0.79 | 0.92 | 0.72* | 0.88 |
| 91 | 2.01 | 2.01 | 50.90 | IPI:IPI00019448.3 | Isoform 1 of Centrosomal protein of 41 kDa | 1 | 1.25 | 1.37 | 1.18 | 0.67 |
| 92 | 2.00 | 51.77 | 75.10 | IPI:IPI00894122.1 | Putative uncharacterized protein APOB | 24 | 0.90 | 0.61* | 0.99 | 0.64* |
| 93 | 2.00 | 2.05 | 47.90 | IPI:IPI00218112.1 | Isoform Short of Glutaryl-CoA dehydrogenase, mitochondrial | 1 | 1.17 | 0.92 | 1.09 | 0.41 |
| 94 | 2.00 | 2.00 | 48.50 | IPI:IPI00299503.2 | Isoform 1 of Phosphatidylinositol-glycan-specific phospholipase D | 1 | 1.26 | 1.43 | 1.12 | 1.11 |
| 95 | 2.00 | 2.00 | 59.70 | IPI:IPI00297550.8 | Coagulation factor XIII A chain | 1 | 1.18 | 1.09 | 1.17 | 1.18 |
| 96 | 2.00 | 2.00 | 57.20 | IPI:IPI00479116.1 | Carboxypeptidase N subunit 2 | 1 | 0.57* | 1.01 | 0.74* | 0.90 |
| 97 | 2.00 | 2.00 | 60.60 | IPI:IPI00792115.1 | Putative uncharacterized protein DKFZp686H17246 | 1 | 0.47 | 0.66 | 0.55 | 1.00 |
| 98 | 2.00 | 2.00 | 56.60 | IPI:IPI00552578.2 | Serum amyloid A protein | 1 | 1.36 | 1.08 | 1.46* | 1.19 |
| 99 | 2.00 | 2.00 | 43.70 | IPI:IPI00004656.3 | Beta-2-microglobulin | 1 | 0.38 | 0.62 | 0.42 | 0.67 |
| 100 | 1.72 | 1.72 | 45.00 | IPI:IPI00010295.1 | Carboxypeptidase N catalytic chain | 1 | 0.83 | 1.07 | 0.77 | 1.33 |
| 101 | 1.72 | 1.72 | 39.90 | IPI:IPI00007240.2 | Coagulation factor XIII B chain | 1 | 1.10 | 1.06 | 1.25 | 1.39* |
| 102 | 1.52 | 1.52 | 57.50 | IPI:IPI00795171.1 | PUS1 42 kDa protein | 1 | 0.78 | 0.87 | 0.78 | 1.08 |
| 103 | 1.45 | 186.57 | 98.20 | IPI:IPI00022434.4 | Putative uncharacterized protein ALB | 328 | 2.08 | 1.36 | 1.65 | 0.58 |
| 104 | 1.42 | 1.49 | 52.70 | IPI:IPI00002579.4 | KDM5B 190 kDa protein | 0 | 1.28 | 1.40 | 1.18 | 0.88 |
| 105 | 1.42 | 1.42 | 48.60 | IPI:IPI00876950.1 | Isoform 2 of Inter-alpha-trypsin inhibitor heavy chain H3 | 0 | 1.06 | 1.47 | 1.47* | 1.47* |
| 106 | 1.38 | 1.40 | 40.10 | IPI:IPI00759542.1 | Isoform 8 of Titin | 0 | 0.97 | 0.94 | 0.92 | 0.85 |
| 107 | 1.30 | 1.30 | 53.50 | IPI:IPI00384481.5 | Tetratricopeptide repeat protein 30B | 1 | 1.41 | 1.07 | 1.22 | 1.08 |

- P<0.05

Down-regulation (The ratios of proteins in disease groups are significantly less than 1.)

- Up-regulation (The ratios of proteins in disease groups are significantly more than 1.)
