# Supplementary material for: Plasma protein profiling of Mild Cognitive Impairment and Alzheimer’s disease using iTRAQ quantitative proteomics
Source: Proteome Sci. 2014 Jan 17;12:5. doi: 10.1186/1477-5956-12-5 (PMC3898732; doi:10.1186/1477-5956-12-5)
Supplement: Additional file 3: Table S3 — The results of total identified proteins of iTRAQ experiment 3. [file 1477-5956-12-5-S3.docx]

Supplementary table 3. The results of total identified proteins of iTRAQ experiment 3

| N | Unused | Total | % Cov | Accession # | Name | Peptide (95%) | aMCI:  Normal | nMCI:  Normal | Normal:  AD | nmdMCI:  Normal |
| --- | --- | --- | --- | --- | --- | --- | --- | --- | --- | --- |
| 1 | 200.73 | 200.73 | 92.7 | IPI:IPI00783987.2 | Complement C3 | 197 | 1.14* | 1.01 | 0.92* | 1.06* |
| 2 | 191 | 191 | 73.9 | IPI:IPI00022229.1 | Apolipoprotein B-100 | 108 | 1.03 | 1.02 | 1.02 | 1.03* |
| 3 | 184.02 | 184.02 | 87.6 | IPI:IPI00478003.1 | Alpha-2-macroglobulin | 241 | 1.13* | 0.99 | 0.90* | 0.99 |
| 4 | 136.26 | 136.26 | 94.4 | IPI:IPI00745872.2 | Isoform 1 of Serum albumin | 303 | 0.60* | 0.62* | 1.30* | 0.75* |
| 5 | 113.09 | 113.12 | 83.4 | IPI:IPI00892604.1 | Complement component C4B (Childo blood group) 2 | 75 | 1.12* | 1.02 | 0.91* | 1.09* |
| 6 | 71.4 | 71.4 | 95.1 | IPI:IPI00298497.3 | Fibrinogen beta chain | 102 | 1.16* | 0.96* | 0.96* | 1.11* |
| 7 | 70.79 | 70.79 | 76.9 | IPI:IPI00021885.1 | Isoform 1 of Fibrinogen alpha chain | 96 | 1.15* | 0.95* | 1.01 | 1.13* |
| 8 | 66 | 66 | 73.1 | IPI:IPI00017601.1 | Ceruloplasmin | 48 | 1.14* | 0.97 | 0.94* | 1.07* |
| 9 | 65.62 | 65.62 | 73.1 | IPI:IPI00029739.5 | Isoform 1 of Complement factor H | 38 | 1.00 | 1.02 | 0.91* | 1.00 |
| 10 | 62.52 | 62.52 | 82.9 | IPI:IPI00877792.1 | Fibrinogen gamma chain | 96 | 1.01 | 0.91* | 0.92* | 1.12* |
| 11 | 60.69 | 60.69 | 71.2 | IPI:IPI00022488.1 | Hemopexin | 69 | 0.93* | 1.06* | 1.03 | 1.01 |
| 12 | 47.64 | 47.64 | 93.6 | IPI:IPI00021841.1 | Apolipoprotein A-I | 59 | 0.66* | 1.03 | 1.45* | 0.85* |
| 13 | 47.09 | 47.09 | 68.2 | IPI:IPI00645038.1 | Inter-alpha (Globulin) inhibitor H2 | 24 | 1.12* | 1.02 | 1.06 | 1.00 |
| 14 | 46.3 | 46.31 | 64.1 | IPI:IPI00019591.2 | Complement factor B | 28 | 0.96 | 0.95* | 0.90* | 0.95 |
| 15 | 44.41 | 44.41 | 77.2 | IPI:IPI00896419.2 | ITIH4 protein | 27 | 0.96 | 0.95 | 0.93* | 0.96 |
| 16 | 43.98 | 44.17 | 64.4 | IPI:IPI00019580.1 | Plasminogen | 25 | 1.17* | 1.04 | 1.05 | 1.07 |
| 17 | 43.85 | 43.85 | 70.5 | IPI:IPI00742696.2 | Vitamin D-binding protein precursor | 30 | 0.68* | 0.98 | 1.07* | 0.80* |
| 18 | 37.36 | 37.36 | 73.3 | IPI:IPI00641737.1 | Haptoglobin-related protein | 23 | 1.07 | 0.96 | 1.01 | 1.16* |
| 19 | 37.01 | 37.01 | 83.3 | IPI:IPI00304273.2 | Apolipoprotein A-IV | 21 | 0.67* | 0.99 | 1.11* | 0.72* |
| 20 | 34.61 | 34.61 | 67.7 | IPI:IPI00032328.2 | Isoform HMW of Kininogen-1 | 23 | 1.16 | 0.98 | 0.91 | 1.13 |
| 21 | 30.36 | 30.36 | 77.5 | IPI:IPI00550991.3 | Alpha-1-antichymotrypsin | 23 | 0.99 | 1.00 | 1.11* | 0.98 |
| 22 | 27.26 | 27.26 | 71.7 | IPI:IPI00022895.7 | Alpha-1B-glycoprotein | 18 | 1.00 | 1.06* | 1.17* | 0.97 |
| 23 | 26.31 | 26.31 | 76.7 | IPI:IPI00019568.1 | Prothrombin (Fragment) | 15 | 1.00 | 1.03 | 1.02 | 0.94 |
| 24 | 26.25 | 26.25 | 71.3 | IPI:IPI00298828.3 | Beta-2-glycoprotein 1 | 18 | 0.80* | 0.97 | 0.87* | 0.95 |
| 25 | 25.29 | 25.29 | 72.5 | IPI:IPI00032179.2 | Antithrombin III variant | 22 | 0.92 | 1.08* | 1.00 | 1.06 |
| 26 | 24.88 | 24.89 | 58.8 | IPI:IPI00292530.1 | Inter-alpha-trypsin inhibitor heavy chain H1 | 13 | 1.24* | 1.06 | 1.05 | 1.13* |
| 27 | 24.38 | 24.38 | 91.8 | IPI:IPI00855916.1 | Transthyretin | 22 | 0.91 | 1.13* | 1.19* | 1.02 |
| 28 | 23.83 | 23.83 | 62.8 | IPI:IPI00926249.1 | AHSG 39 kDa protein | 24 | 0.97 | 1.01 | 0.99 | 1.00 |
| 29 | 21.44 | 21.44 | 57 | IPI:IPI00022371.1 | Histidine-rich glycoprotein | 12 | 0.87* | 0.95 | 0.96 | 0.92 |
| 30 | 21.08 | 21.09 | 49.2 | IPI:IPI00032291.2 | Complement C5 | 8 | 1.00 | 0.98 | 0.99 | 0.99 |
| 31 | 20.7 | 20.7 | 56 | IPI:IPI00022395.1 | Complement component C9 | 10 | 1.11 | 1.00 | 0.96 | 1.02 |
| 32 | 19.85 | 19.85 | 78.6 | IPI:IPI00884926.1 | Orosomucoid 1 precursor | 43 | 0.59* | 1.00 | 1.07* | 0.75* |
| 33 | 18.94 | 18.94 | 51.3 | IPI:IPI00298971.1 | Vitronectin | 15 | 0.96 | 1.00 | 1.01 | 0.93* |
| 34 | 18.66 | 18.66 | 50.9 | IPI:IPI00021727.1 | C4b-binding protein alpha chain | 8 | 0.85* | 0.80* | 0.82* | 0.87* |
| 35 | 18.65 | 18.65 | 64.3 | IPI:IPI00026314.1 | Isoform 1 of Gelsolin | 9 | 1.15* | 0.95 | 0.95 | 1.09* |
| 36 | 18.07 | 18.07 | 65.5 | IPI:IPI00400826.1 | Clusterin isoform 1 | 9 | 0.81* | 0.92* | 1.14* | 0.84* |
| 37 | 17.23 | 17.23 | 59.6 | IPI:IPI00291866.5 | Plasma protease C1 inhibitor | 12 | 0.85 | 0.94 | 1.02 | 0.99 |
| 38 | 16.66 | 16.66 | 56 | IPI:IPI00022426.1 | Protein AMBP | 10 | 1.10 | 0.99 | 0.93 | 0.98 |
| 39 | 14.89 | 14.89 | 60.9 | IPI:IPI00292950.4 | Serpin peptidase inhibitor, clade D (Heparin cofactor), member 1 | 7 | 1.00 | 0.97 | 1.00 | 0.95 |
| 40 | 13.56 | 13.56 | 79 | IPI:IPI00021854.1 | Apolipoprotein A-II | 23 | 0.53* | 1.06 | 0.98 | 0.71* |
| 41 | 13.46 | 13.46 | 68.3 | IPI:IPI00480192.1 | Retinol binding protein 4, plasma | 10 | 1.10 | 1.09* | 1.03 | 1.11* |
| 42 | 12.97 | 12.97 | 37.1 | IPI:IPI00845263.1 | fibronectin 1 isoform 2 preproprotein | 7 | 0.97 | 0.74* | 1.16* | 1.28* |
| 43 | 11.83 | 11.83 | 38.6 | IPI:IPI00879709.3 | Complement component 6 precursor | 5 | 1.09 | 1.07 | 1.07 | 1.07 |
| 44 | 11.16 | 11.16 | 56.5 | IPI:IPI00022391.1 | Serum amyloid P-component | 4 | 1.03 | 0.97 | 0.98 | 0.93 |
| 45 | 10.96 | 11.79 | 53.9 | IPI:IPI00019943.1 | Afamin | 4 | 0.69* | 0.96 | 1.01 | 0.87* |
| 46 | 10.02 | 10.02 | 59.4 | IPI:IPI00032220.3 | Angiotensinogen | 6 | 1.20 | 1.07 | 1.07 | 1.14 |
| 47 | 10 | 13.77 | 75.1 | IPI:IPI00020091.1 | Alpha-1-acid glycoprotein 2 | 12 | 0.78 | 1.04 | 1.09 | 1.01 |
| 48 | 9.44 | 9.44 | 51.8 | IPI:IPI00218732.3 | Serum paraoxonase/arylesterase 1 | 5 | 1.08 | 1.02 | 1.06 | 1.06 |
| 49 | 9.29 | 9.3 | 55.5 | IPI:IPI00294395.1 | Complement component C8 beta chain | 4 | 0.97 | 0.98 | 0.89 | 0.97 |
| 50 | 9.15 | 9.15 | 51 | IPI:IPI00296165.6 | Complement C1r subcomponent | 3 | 1.03 | 0.97 | 0.84 | 0.96 |
| 51 | 8.69 | 8.69 | 54.8 | IPI:IPI00879231.1 | Alpha-2-antiplasmin | 4 | 0.86* | 1.21* | 1.05 | 0.96 |
| 52 | 8.61 | 8.61 | 39.2 | IPI:IPI00019581.1 | Coagulation factor XII | 4 | 1.01 | 0.97 | 0.98 | 0.95 |
| 53 | 8.57 | 8.57 | 72.6 | IPI:IPI00021842.1 | Apolipoprotein E | 5 | 0.85 | 1.04 | 1.00 | 0.87 |
| 54 | 7.9 | 7.9 | 38.8 | IPI:IPI00291867.3 | Complement factor I | 4 | 1.17 | 1.11 | 1.04 | 1.35* |
| 55 | 7.72 | 7.72 | 38.2 | IPI:IPI00020986.2 | Lumican | 4 | 0.95 | 0.92* | 0.93 | 1.01 |
| 56 | 7.38 | 7.38 | 58.6 | IPI:IPI00329775.7 | Isoform 1 of Carboxypeptidase B2 | 3 | 0.89 | 1.00 | 0.92 | 1.05 |
| 57 | 7.19 | 7.19 | 47.7 | IPI:IPI00166729.4 | Alpha-2-glycoprotein 1, zinc | 3 | 0.76 | 1.00 | 1.01 | 0.84 |
| 58 | 7.1 | 7.1 | 64.1 | IPI:IPI00022394.2 | Complement C1q subcomponent subunit C | 3 | 1.49* | 1.17* | 1.11 | 1.23 |
| 59 | 6.78 | 6.78 | 55.5 | IPI:IPI00293925.2 | Isoform 1 of Ficolin-3 | 3 | 1.14 | 1.16* | 0.90 | 1.04 |
| 60 | 6.64 | 6.64 | 41.1 | IPI:IPI00643948.2 | Complement component 1, q subcomponent, B chain | 7 | 1.14 | 1.04 | 1.20* | 1.05 |
| 61 | 6.41 | 6.41 | 38.7 | IPI:IPI00296608.6 | Complement component C7 | 3 | 1.25 | 1.46 | 1.11 | 1.15 |
| 62 | 6.33 | 6.33 | 47.8 | IPI:IPI00006114.4 | Pigment epithelium-derived factor | 3 | 1.12 | 0.86 | 0.82 | 1.03 |
| 63 | 6.13 | 6.15 | 47.8 | IPI:IPI00654888.4 | Plasma kallikrein | 3 | 0.88 | 0.91 | 0.90 | 0.98 |
| 64 | 6.07 | 6.07 | 40 | IPI:IPI00479708.5 | IGHM protein | 3 | 1.41* | 1.00 | 1.47* | 1.12* |
| 65 | 6 | 6 | 38.2 | IPI:IPI00163207.1 | Isoform 1 of N-acetylmuramoyl-L-alanine amidase | 5 | 0.89 | 1.01 | 0.98 | 1.04 |
| 66 | 6 | 6 | 58.8 | IPI:IPI00022392.1 | Complement C1q subcomponent subunit A | 3 | 1.08 | 0.84 | 0.66 | 0.91 |
| 67 | 5.3 | 5.3 | 44 | IPI:IPI00873445.1 | PROS1 80 kDa protein | 3 | 0.74* | 0.80* | 0.99 | 0.86 |
| 68 | 4.57 | 4.57 | 53.4 | IPI:IPI00006662.1 | Apolipoprotein D | 2 | 0.71* | 1.05 | 1.06 | 0.79* |
| 69 | 4.35 | 4.35 | 74.6 | IPI:IPI00019399.1 | Serum amyloid A-4 protein | 1 | 0.94 | 1.01 | 0.89 | 0.92* |
| 70 | 4.05 | 17.49 | 79.9 | IPI:IPI00844156.2 | SERPINC1 protein | 14 | 0.90 | 1.12 | 1.41* | 0.98 |
| 71 | 4.02 | 4.02 | 35.2 | IPI:IPI00853068.1 | Alpha 2 globin variant (Fragment) | 3 | 0.76* | 0.91 | 0.79* | 0.85 |
| 72 | 4 | 4 | 31.4 | IPI:IPI00027235.1 | Isoform 1 of Attractin | 2 | 1.08 | 1.02 | 1.17 | 1.10 |
| 73 | 4 | 4 | 49.9 | IPI:IPI00328609.3 | Kallistatin | 2 | 1.16 | 0.92 | 0.99 | 0.95 |
| 74 | 3.3 | 3.3 | 46.5 | IPI:IPI00009028.1 | Tetranectin | 2 | 1.31 | 1.12 | 1.00 | 1.08 |
| 75 | 3.11 | 3.11 | 51.5 | IPI:IPI00011261.2 | Complement component C8 gamma chain | 1 | 1.32 | 1.05 | 1.16 | 1.06 |
| 76 | 3.1 | 3.1 | 45.5 | IPI:IPI00021856.3 | Apolipoprotein C-II | 1 | 0.42* | 0.70 | 0.97 | 0.56* |
| 77 | 2.92 | 2.92 | 40 | IPI:IPI00017696.1 | Complement C1s subcomponent | 1 | 1.09 | 0.92 | 0.90 | 1.04 |
| 78 | 2.57 | 2.57 | 47 | IPI:IPI00514475.5 | Isoform 1 of Apolipoprotein L1 | 1 | 0.55 | 0.85 | 1.43 | 0.63 |
| 79 | 2.48 | 2.48 | 36.3 | IPI:IPI00011252.1 | Complement component C8 alpha chain | 1 | 1.16 | 1.09 | 0.86* | 1.39* |
| 80 | 2.46 | 2.46 | 78.2 | IPI:IPI00004656.3 | Beta-2-microglobulin | 1 | 0.90 | 0.96 | 0.99 | 1.01 |
| 81 | 2.35 | 25.62 | 57.2 | IPI:IPI00025426.2 | Isoform 1 of Pregnancy zone protein | 26 | 1.05 | 1.01 | 0.98 | 0.99 |
| 82 | 2.35 | 2.46 | 18.2 | IPI:IPI00746623.2 | Hyaluronan-binding protein 2 | 1 | 1.11* | 0.84 | 0.84* | 1.19* |
| 83 | 2.34 | 2.38 | 48.1 | IPI:IPI00006154.1 | Isoform Long of Complement factor H-related protein 2 | 1 | 0.90 | 0.87 | 1.16 | 0.86 |
| 84 | 2.34 | 2.34 | 50.3 | IPI:IPI00884107.1 | Beta-globin gene from a thalassemia patient | 1 | 0.62* | 0.80* | 0.91 | 0.77* |
| 85 | 2.31 | 2.31 | 20.1 | IPI:IPI00218803.3 | Isoform B of Fibulin-1 | 1 | 1.19 | 1.15 | 1.06 | 1.03 |
| 86 | 2.04 | 3.2 | 37.3 | IPI:IPI00292946.1 | Thyroxine-binding globulin | 1 | 1.23 | 1.06 | 0.95 | 1.05 |
| 87 | 2.03 | 2.86 | 56.6 | IPI:IPI00303963.1 | Complement C2 | 1 | 1.06 | 0.99 | 1.06 | 1.13 |
| 88 | 2.01 | 2.01 | 42.5 | IPI:IPI00026199.2 | Glutathione peroxidase 3 | 1 | 0.64* | 0.78 | 0.72* | 0.67* |
| 89 | 2 | 2 | 55.5 | IPI:IPI00925635.1 | Insulin-like growth factor binding protein, acid labile subunit isoform 1 precursor | 1 | 1.20 | 1.16 | 1.17 | 1.00 |
| 90 | 2 | 2 | 50.4 | IPI:IPI00657670.1 | Apolipoprotein C-III variant 1 | 22 | 0.80 | 0.92 | 0.94 | 0.77 |
| 91 | 1.93 | 34.32 | 78.2 | IPI:IPI00797833.3 | Kininogen 1 | 1 | 1.11 | 0.93 | 0.87 | 0.95 |
| 92 | 1.7 | 1.7 | 34.5 | IPI:IPI00479116.1 | Carboxypeptidase N subunit 2 | 282 | 0.91 | 0.89 | 0.74 | 1.06 |
| 93 | 1.57 | 129.49 | 93.3 | IPI:IPI00022434.4 | Putative uncharacterized protein ALB | 1 | 1.54 | 1.47 | 1.44 | 1.42* |
| 94 | 1.4 | 1.4 | 51.9 | IPI:IPI00027790.3 | AMMECR1-like protein | 1 | 1.37 | 0.75 | 0.43 | 1.12 |
| 95 | 1.4 | 1.4 | 78.8 | IPI:IPI00152452.1 | Uncharacterized protein C8orf16 | 1 | 0.99 | 2.02 | 1.39* | --- |
| 96 | 1.31 | 1.32 | 36.3 | IPI:IPI00028413.8 | Isoform 1 of Inter-alpha-trypsin inhibitor heavy chain H3 |  | 1.08 | 1.09 | 1.08 | 1.05 |

* P<0.05

Down-regulation (The ratios of proteins in disease groups are significantly less than 1.)

Up-regulation (The ratios of proteins in disease groups are significantly more than 1.)
